# The phylogeny of C/S1 bZIP transcription factors reveals a shared algal ancestry and the pre-angiosperm translational regulation of S1 transcripts

**DOI:** 10.1038/srep30444

**Published:** 2016-07-26

**Authors:** Alessia Peviani, Jeroen Lastdrager, Johannes Hanson, Berend Snel

**Affiliations:** 1 Theoretical Biology and Bioinformatics, Department of Biology, Faculty of Science, Utrecht University, Utrecht, The Netherlands; 2 Molecular Plant Physiology, Institute of Environmental Biology, Utrecht University, Utrecht, The Netherlands; 3 Umeå Plant Science Centre, Department of Plant Physiology, Umeå University, Umeå, Sweden

## Abstract

Basic leucine zippers (bZIPs) form a large plant transcription factor family. C and S1 bZIP groups can heterodimerize, fulfilling crucial roles in seed development and stress response. S1 sequences also harbor a unique regulatory mechanism, termed Sucrose-Induced Repression of Translation (SIRT). The conservation of both C/S1 bZIP interactions and SIRT remains poorly characterized in non-model species, leaving their evolutionary origin uncertain and limiting crop research. In this work, we explored recently published plant sequencing data to establish a detailed phylogeny of C and S1 bZIPs, investigating their intertwined role in plant evolution, and the origin of SIRT. Our analyses clarified C and S1 bZIP orthology relationships in angiosperms, and identified S1 sequences in gymnosperms. We experimentally showed that the gymnosperm orthologs are regulated by SIRT, tracing back the origin of this unique regulatory mechanism to the ancestor of seed plants. Additionally, we discovered an earlier S ortholog in the charophyte algae *Klebsormidium flaccidum*, together with a C ortholog. This suggests that C and S groups originated by duplication from a single algal proto-C/S ancestor. Based on our observations, we propose a model wherein the C/S1 bZIP dimer network evolved in seed plants from pre-existing C/S bZIP interactions.

The basic leucine zipper (bZIP) family of transcription factors is one of the largest in plants, represented in angiosperms by 13 subfamilies involved in the regulation of fundamental physiological and developmental processes^1^,^2^. The participation of several members in the response to environmental cues makes this transcription factor family a promising research subject for crop yield and stress resistance improvement, motivating the recent burst of genome-wide bZIP analyses in a variety of cultivated species^3^,^4^,^5^,^6^,^7^,^8^,^9^,^10^,^11^,^12^.

Within the S bZIP subfamily, the group of orthologs named S1^13^ is specifically involved in seed development and metabolic reprogramming in response to stress^14^,^15^,^16^,^17^,^18^,^19^, probably downstream of SnRK1 kinase signaling^20^,^21^. The specificity of S1 bZIP activity in low energy conditions is achieved via a unique regulatory mechanism, termed Sucrose-Induced Repression of Translation (SIRT)^22^,^23^,^24^, which relies on the presence of a characteristic uORF at the 5′ leader of the bZIP transcript (5′uORF). According to the current SIRT model, high sugar availability increases the affinity of the ribosome for the 5′uORF, possibly triggering its translation into a Sucrose Control (SC) peptide, and preventing protein synthesis at the bZIP main ORF (mORF)^24^,^25^. While the SC-peptide remains to be isolated, key amino acid residues and other 5′uORF features appear necessary for SIRT to take place, and accordingly are conserved across S1 bZIP orthologs^24^. Importantly, SIRT is considered a characterizing feature of these transcription factors, as no similar uORF sequences or uORF-based repression mechanism by sucrose has been observed in other bZIP groups, or more generally in other plant gene families^26^.

A further layer of regulation is achieved through the formation of heterodimers between S1 and C bZIP subfamily members, also characterized as regulators in seed development^15^,^27^ and stress response^28^. The interaction between S1 and C bZIP sequences relies on the compatibility of their bZIP domains^29^, likely as a consequence of the close phylogenetic relationship between C and S subfamilies; indeed it has been proposed that S subfamily emerged from the C subfamily in an angiosperm-specific duplication event^2^. Remarkably, while other bZIP subfamilies are mainly involved in simple homo- or quasi-homodimerization events (i.e. dimerization between close paralogs)^30^, C/S1 interactions give rise to a more complex dimerization network, as shown in the model species arabidopsis (*Arabidopsis thaliana*)^13^,^14^. Moreover, C and S1 bZIP members interact specifically while avoiding dimerization with other S sequences^13^,^27^, indicating the presence of selective pressure to prevent promiscuity.

The functional importance of C/S1 bZIP heterodimers for transcriptional activity was reported in a series of double overexpression experiments, which showed a strong synergistic effect on both up- and down-regulation of selected targets^15^,^27^,^31^. At the same time, the lack of drastic phenotypes in loss-of-function mutants indicates that the system allows for a certain degree of redundancy^18^,^19^,^32^. These observations, together with the individual C and S1 bZIPs interaction preferences^13^, tissue- and condition-dependent expression patterns^16^, translational regulation by SIRT^24^, and phosphorylation of C group members^33^,^34^,^35^, seem to allow for a signal integration system endowed with tremendous flexibility in the fine-tuning of target genes. It has been proposed that such system could help optimizing the effectiveness of stress response depending on the specific environmental threat^36^. However, experimental studies of heterodimerization preferences and other bZIP-specific properties such as SIRT are still limited to a handful of dicots^13^,^24^,^37^,^38^,^39^,^40^.

Phylogenetics represents a powerful resource to assess the C/S1 bZIP transcription factor network conservation across plant species, providing a framework for the transfer of functional information between model organisms and crops. Unfortunately, while the identification of C bZIP orthologs appears relatively straightforward, putative S1 sequences proved troublesome in previous phylogenetic studies^2^. Duplications within the S subfamily gave rise to the largest and most diverse group among bZIP transcription factors, hindering the assessment of orthology relationships between plant lineages. As a consequence, while sequence alignments based on simple similarity searches indicate the presence of 5′uORF-bearing S1 orthologs in both dicots and monocots^24^,^39^,^41^, advanced multi-species phylogenetic reconstructions are still unable to reconcile these observations into a comprehensive phylogeny of angiosperm S1 bZIPs^2^. The identification and study of S1 bZIP orthologs in novel species would greatly benefit from a solid phylogeny-based classification reference.

A high-resolution phylogeny yielding reliable ortholog identification could also clarify the early evolution of the C/S1 bZIP transcription factor network, which remains unexplored. Currently, we can extrapolate that heterodimer formation was not possible before the emergence of flowering plants, as the first C and S bZIP representatives were reported in bryophytes and angiosperms, respectively^2^. However, the limited quantity and quality of plant sequencing data available at that time^2^ suggests that this might be an incomplete picture. The accurate characterization of both C and S bZIP early members, and especially of the younger S subfamily, is therefore crucial to generate sensible hypotheses on the dimerization network origin.

Recently, a plethora of plant genome projects made available valuable sequence information for comparative studies, not only among crops, but also in poorly characterized early branching lineages^42^,^43^,^44^,^45^,^46^,^47^,^48^,^49^,^50^,^51^,^52^. By taking advantage of this resource, we investigated the intertwined role of the C/S1 bZIP transcription factor network members during plant evolution. The new data allowed us to resolve S subfamily classification in angiosperms, finally confirming the presence of S1 bZIP orthologs in both dicots and monocots. The analysis of C bZIP orthologs also clarified the pattern of gene duplications and losses in flowering plants, providing additional information for comparative studies. Surprisingly, our results showed S subfamily to be much older than previously thought, with basal S sequences discovered before the emergence of land plants, and possibly originating at the same time as the C subfamily. We additionally proved the presence of S1 bZIPs in the common ancestor of spermatophytes, as supported by *in vivo* experiments confirming regulation of putative S1 orthologs from gymnosperms by SIRT. Finally, we outlined new hypotheses on the origin and specialization of the C/S1 bZIP dimerization network during plant evolution, discussing in particular its putative emergence from pre-existing C/S interactions.

## Results

### C and S1 bZIP subfamilies show lineage-specific patterns of gene duplications and losses in angiosperms

**Figure 1:**
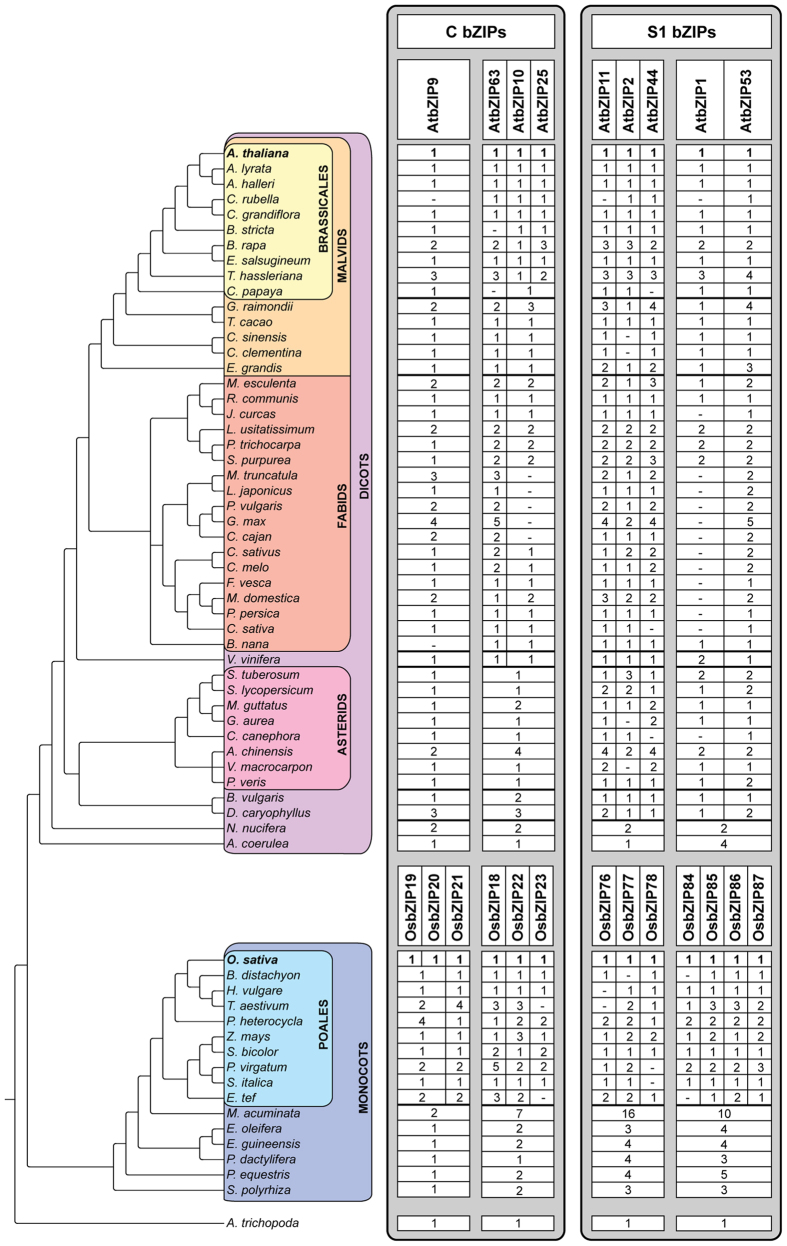
Conservation of C and S1 bZIP orthologs across angiosperm species. The figure combines consistent topologies from independent lineage-specific Maximum Likelihood phylogenetic trees of angiosperm C and S1 bZIP subfamilies. For each species, values indicate the number of orthologs directly related to arabidopsis (*A. thaliana)* or rice (*O. sativa*) as reference bZIPs (in bold) for dicots or monocots, respectively. Notice that no direct correspondence exists between individual dicot and monocot orthologs (individual columns), but only between groups of orthologs (white blocks). Dashes indicate missing orthologs. See Suppl. Figs S1 and S2 for original C and S1 bZIP trees, respectively.

The C/S1 bZIP transcription factor network is still poorly characterized in non-model species. Here we analyze a large set of recently published plant genomes to thoroughly assess C and S1 bZIP transcription factors conservation across flowering plants, and in particular S1 bZIP ortholog relationships between dicots and monocots. First, we assembled a database of sequenced angiosperm species encompassing previously unexplored lineages, such as basal angiosperms, asterids, and non-poales monocots (Suppl. Table S1). C and S1 orthlogs were then collected through iterative BLAST and HMMER searches using reference bZIPs as queries, and aligned to generate phylogenetic trees of each subfamily (see Methods and Suppl. Figs S1 and S2). The combined results of independent reconstructions for different ortholog groups and plant lineages are shown in Fig. 1.

**Figure 2:**
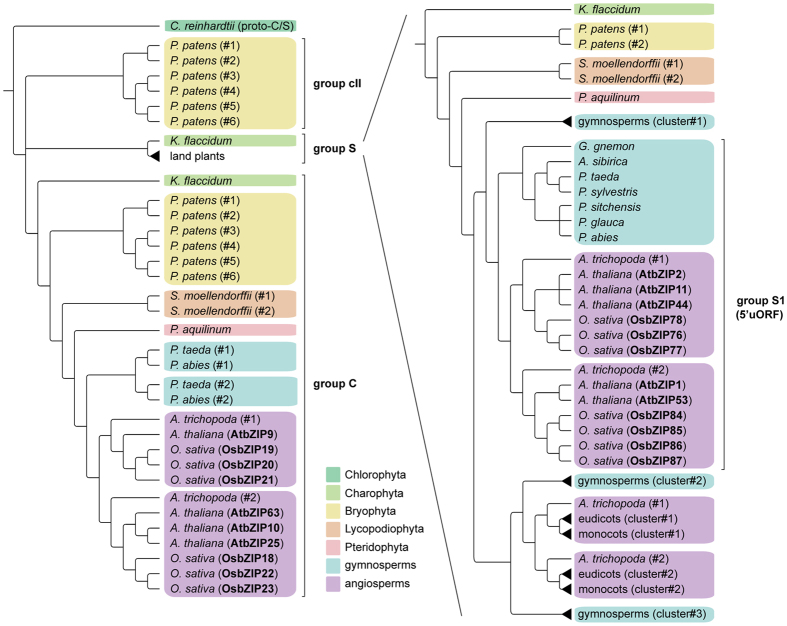
A phylogeny of C and S bZIP subfamilies in green plants. Simplified phylogenetic trees of the C and S bZIP subfamilies showing the relationship between the two groups of orthologs in green plants. Known C and S1 bZIP orthologs from arabidopsis and rice are shown in bold. The topology represents the consensus of independent phylogenetic reconstructions shown in Suppl. Fig. S3. S1 bZIP orthologs are indicated based on the presence of conserved S1 5′uORFs.

Our phylogenetic trees showed a clear subdivision of S1 bZIPs into two further groups of orthologs, each conserved between dicots and monocots as showed by consistent topologies between several independent lineage-specific reconstructions (Fig. 1 and Suppl. Fig. S2). The first identified group included AtbZIP2, AtbZIP11, and AtbZIP44 from the model dicot arabidopsis (*Arabidopsis thaliana),* and OsbZIP76, OsbZIP77, and OsbZIP78 from the representative monocot rice (*Oryza sativa*), while the second group included AtbZIP1 and AtbZIP53, and OsbZIP84, OsbZIP85, OsbZIP86, and OsbZIP87 from the same two species, respectively. Orthology between dicot and monocot sequences from each group can be described as a “many-to-many” relationship, as further within-group duplications appear independent between the two plant lineages (Fig. 1; see also Fig. 2). The duplication which created these two groups of orthologs likely occurred early in angiosperm evolution, as we found two S1 bZIP genes in the amborella (*Amborella trichopoda*) genome. These sequences clustered consistently with either S1 bZIP ortholog group in most lineage-specific trees, although with low bootstrap support values (see Suppl. Fig. S2).

The C bZIP subfamily is divided in two main groups of orthologs: one represented by arabidopsis AtbZIP9, and the other by AtbZIP10, AtbZIP25, and AtbZIP63. The corresponding sequences for each group in rice were OsbZIP19, OsbZIP20, and OsbZIP21, and OsbZIP18, OsbZIP22, and OsbZIP23, respectively (Fig. 1; see also Fig. 2 and Suppl. Fig. S1). The basal angiosperm amborella also harbors two C bZIP sequences, with strongly supported membership in either C ortholog group (Fig. 1 and Suppl. Fig. S1). In more recent angiosperm lineages, the second group of orthologs might be further split into two separate clusters: one including AtbZIP63 and OsbZIP19, and the other collecting AtbZIP10 and AtbZIP25, and OsbZIP20 and OsbZIP21, as previously proposed^2^. Importantly, our analyses allowed us to detect unexpected patterns of gene duplications and losses in both C and S1 bZIP phylogeny (Fig. 1 and Suppl. Figs S1 and S2). For instance, C subfamily AtbZIP10 and AtbZIP25 turned out to be the result of a gene duplication event specific to brassicales, constituting in fact a lineage-specific pair of paralogs (Fig. 1). Other eudicots on the contrary seem to possess a depleted set of orthologs for these sequences; we observed this in the subgroup of fabales termed faboideae, which includes soybean (*Glycine max*), and in the early branching eudicots *Aquilegia coerulea, Vitis vinifera*, and asterids (Fig. 1 and Suppl. Fig. S1). Absence in these latter species might be potentially explained by a poor assignment of their AtbZIP63 orthologs, which could be ancestral not only to AtbZIP63 but also to AtbZIP10 and AtbZIP25; however, our phylogenetic reconstruction points more convincingly to a secondary gene loss (see Suppl. Fig. S1).

Among S1 bZIPs, we revealed the complete lack of AtbZIP1 orthologs in the eudicot groups fabales (soybean, *Cajanus cajan*, *Lotus japonicus*, *Medicago truncatula*, *Phaseolus vulgaris*), cucurbitales (*Cucumis melo* and *C. sativus*), and rosales (*Cannabis sativa*, *Fragaria vesca*, *Malus domestica*, *Prunus persica*), which include many species of agricultural importance. This is likely due to a secondary gene loss in the ancestor of these lineages, as AtbZIP1 orthologs are found in other fabids and in their sister lineage malvids. As a consequence, direct S1 orthologs comparisons with lineages branching before *V. vinifera*, such as solanales (*Solanum lycopersicum* and *S. tuberosum*) among asterids, should be considered carefully. A similar situation is present in monocots: while poales could be easily compared with rice reference sequences, species that diverged early on (e.g. *Musa acuminata*) show an independent pattern of duplications for each of the C and S1 ortholog subgroups (Fig. 1 and Suppl. Figs S1 and S2).

The simple scheme presented here (Fig. 1) condenses the information from the fully detailed phylogeny available in the Supplementary Material. Together, our results illustrate the important role of phylogenetics for reliable ortholog identification, especially in the case of gene families shaped by multiple duplication and loss events, such as S1 bZIPs.

### C and S bZIPs originated as sister groups before the emergence of land plants

Similar to the recent phylogenetic history of C and S1 bZIPs in angiosperms, the early evolution of C and S groups was also compromised by species sampling^2^. We included in our analyses a larger set of early branching species, encompassing the multicellular algae *Klebsormidium flaccidum* (charophyte), the bryophyte *Physcomitrella patens*, the spikemoss *Selaginella moellendorffii* (lycopodiophyte), and several pteridophyte and gymnosperm species (see Suppl. Table S1). For the identification of C and S sequences we adopted the same strategy used for angiosperms C and S1 bZIPs (see Methods), additionally including amborella, rice, and arabidopsis C and S bZIP sequences as flowering plants representatives in the phylogenetic reconstructions.

Our analyses resulted in the discovery of unambiguous C and S sequences from gymnosperms to charophytes (Fig. 2). For the S subfamily in particular, our findings completely abolish the current view of these sequences as an angiosperm-specific innovation. Previous analyses described two additional groups of sequences related to C and S bZIPs in early branching plant lineages, named cI and cII^2^. Our analyses identified all of the previously reported cI subfamily members as S class orthologs, therefore our results make the definition of cI subfamily obsolete. Subfamily cII, as described later, was instead recovered as a separate ortholog group in our analyses. S sequences from more ancestral species did not show any tendency to cluster specifically with S1 or any other S group of orthologs from seed plants (Fig. 2), indicating that these groups emerged from spermatophyte-specific duplications. Importantly, the discovery of an S bZIP sequence in the charophyte *K. flaccidum* creates a tremendous gap between the first appearance of an S subfamily member and the later expansion of the group, indicating these transcription factors are likely endowed with more ancestral functions than previously thought.

*K. flaccidum* was also the earliest diverging species to harbor a C bZIP sequence in our phylogenetic reconstruction, bringing back the origin of the subfamily from bryophytes^2^ to charophytes, and therefore from after to before the land colonization event. Importantly, the discovery of both C and S earliest known members in a charophyte (Fig. 2) is an unprecedented clue to the origin of these subfamilies by duplication from a shared ancestor, and suggests a possible role of both subfamilies in the later emergence of land plants.

In addition to C and S orthologs, we found a third group of sequences in bryophytes, corresponding to previously identified cII bZIPs^2^. In our analyses, cII bZIPs were missing in charophytes and vascular plant species, confirming this group is bryophyte-specific (Fig. 2). Finally, chlorophytes harbored a more ancestral type of sequences, termed “proto-C”^2^, which we deemed appropriate to rename “proto-C/S” in order to reflect its parental relationship to both C and S subfamilies (Fig. 2 and Suppl. Fig. S3).

Surprisingly, among the S bZIPs identified in gymnosperms we observed a group of sequences clustering together with angiosperms S1 bZIPs, suggesting they might be S1 orthologs (Fig. 2 and Suppl. Fig. S3). Previous phylogenetic analyses indicated S1 bZIP transcription factors as a recent innovation in plant evolution, likely restricted to angiosperms as the rest of the S subfamily^2^. However, our discovery of putative S1 orthologs in both gymnosperms and angiosperms indicates the possible origin of S1 bZIPs in the ancestor of spermatophytes. We also found a putative S1 ortholog from *Pteridium aquilinum*, but the interpretation is less reliable as we did not observe members in other fern species. Rather than an angiosperm-specific innovation^2^, S1 bZIPs might therefore be regarded as a common toolkit of seed plants, which potentially contributed to the radiation of the entire lineage.

### *In vivo* reporter gene assays confirm 5′uORF-mediated SIRT in gymnosperms S1 bZIP orthologs

Our discovery of putative S1 bZIP members in gymnosperms prompted us to assess the conservation of SIRT-mediating 5′uORFs in these species. Given their ancestral relationship to the entire group of angiosperm S bZIPs, we also hypothesized that the S sequences from early branching species might harbor 5′uORFs with similar properties. Therefore we extracted the upstream region of each identified S (including putative gymnosperm S1) sequence from our genomic database, whenever available, and searched for the presence of 5′uORFs similar to those observed in arabidopsis and rice S1 bZIP 5′UTRs (see Methods).

**Figure 3:**
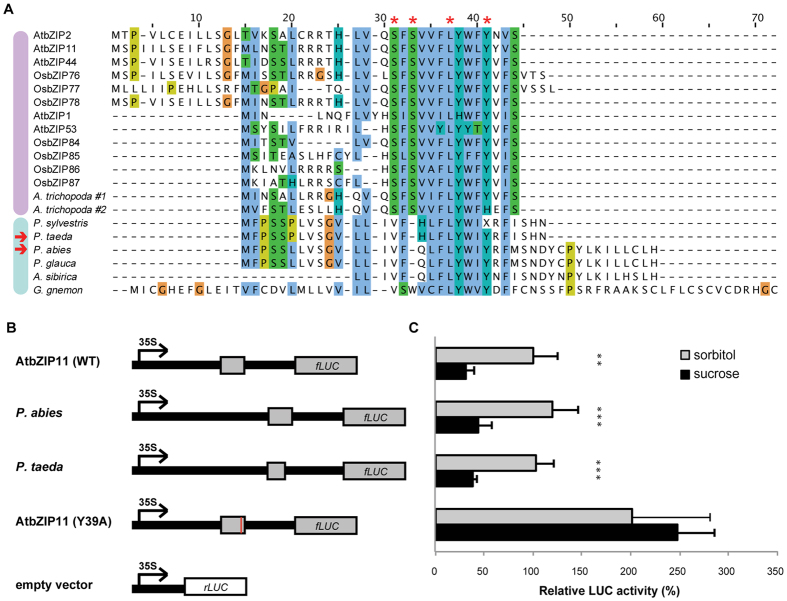
Sequence alignment of 5′uORFs and experimental confirmation of SIRT from gymnosperms putative S1 bZIPs. (**A**) Alignment of 5′uORFs from angiosperms and gymnosperms S1 bZIPs showing conservation across each lineage (indicated in violet and light blue respectively, according to the color code of Fig. 2). Residues known to be necessary for SIRT in arabidopsis^24^ are marked with a red star. Gymnosperms sequences tested for SIRT are indicated with a red arrow. (**B**) Schematic representation of the constructs used to test SIRT in the transient LUC expression assay. Rectangles represent 5′uORFs shown in panel A, with proportions reflecting length and distance from the main ORF. A red line in the mutant AtbZIP11 (Y39A) indicates the substitution point (negative control). An empty 35S:rLUC construct was used to normalize LUC activity data. (**C**) Results of relative LUC activity assays. Normalized LUC activity is presented relative to arabidopsis WT construct results in sorbitol. Values represent the average of at least four biological replicates. Error bars indicate SD from the mean. Stars indicate the significance of a two-tailed distribution t-test with unequal variance (**p < 0.01, ***p < 0.005). For the original results see Suppl. Table S2.

Our results showed matching 5′uORFs in candidate S1 bZIPs from most gymnosperms (Fig. 3A); however, no hit was found in other S orthologs from these species, nor in S subfamily representatives from more early branching plants. Thus, this feature was not inherited by S1 bZIPs from more ancestral S sequences. The newly discovered gymnosperm 5′uORFs shared with angiosperms only two of the four residues thought to be essential for S1 bZIP regulation^24^, i.e. Leu-35 and Tyr-39 from arabidopsis bZIP11, while Ser-29 and Ser-31 were not conserved (Fig. 3A). The termination codon position, another invariable feature of angiosperm S1 5′uORFs^24^, was also different in gymnosperm species (Fig. 3A). Still, the extent of sequence conservation within gymnosperms themselves appears striking, suggesting functionality (Fig. 3A).

To test the ability of gymnosperm S1 5′uORFs to mediate SIRT, we proceeded with specific experimental assays. For the test we selected two representative sequences, from *Picea abies* and *Pinus taeda*, based on their quality and completeness (see sequences in Fig. 3A). The wild type (WT) AtbZIP11 5′uORF was included as a positive control, and the SIRT loss of function mutant (Y39A) was included as a negative control. The effect of each sequence on translational regulation was then tested in a transient luciferase (LUC) expression assay in the presence of either sucrose or sorbitol, the latter serving as an osmotic control (see Methods).

Our results showed that both *P. abies* and *P. taeda* S1 5′uORF sequences could efficiently mediate the translational repression of the LUC reporter gene in the presence of sucrose (Fig. 3B,C and Suppl. Table S2). The magnitude of the effect was significantly higher than the SIRT loss of function mutant (Y39A), and in fact similar to that observed for WT AtbZIP11, i.e. more than 2-fold decrease in expression at the given experimental conditions with little variation between replicates (Fig. 3C and Suppl. Table S2). Thus, we confirmed that the newly found gymnosperm S1 5′uORFs are capable of mediating SIRT.

The conservation of SIRT in gymnosperm S1 sequences might reflect their involvement in the metabolic adaptation to low energy conditions (i.e. sucrose depletion), as observed for angiosperm orthologs, and the need for downscaling their activity when energy levels are restored. Importantly, this is the first time SIRT is experimentally reported for not non-dicot sequences, extending the relevance of this regulatory mechanism to distant plant lineages. In particular, our discovery indicates that SIRT-regulated S1 orthologs were present in the common ancestor of spermatophytes, and that monocot 5′uORFs found through previous similarity searches^24^,^26^ are likely to be functional. Moreover, we suggest that an in-depth comparison of the newly identified gymnosperm sequences with known angiosperms S1 5′uORFs might help clarifying the mechanistic details of SIRT. For instance, it is unclear whether 5′uORF-encoded SC peptides sense sugar molecules directly, or through association to a more complex regulatory machinery. Notably, our assays of gymnosperm sequences took place in arabidopsis cells (see Methods), exploiting the molecular machinery available in this species, and successfully reproduced SIRT in spite of significant differences with angiosperm 5′uORF-encoded peptides, i.e. in the conservation of two specific amino acid residues and the C-terminal position. Our results might therefore indicate that the SIRT mechanism relies on structural conformation rather than on the recognition of specific sequence motifs.

## Discussion

In this work, we presented an integrated phylogenetic reconstruction of the C/S1 bZIP transcription factor network members across plant evolution. In a previous publication^2^, which at the time represented the most comprehensive study of plant bZIPs available, the phylogenetic details of the S subfamily were particularly elusive, limiting the investigation of this regulatory system. Here we finally confirmed the presence of S1 orthologs in both eudicots and monocots, providing phylogenetic evidence for previous observations based on simple sequence similarity searches^24^,^39^,^41^. Moreover, we were able to identify SIRT-mediating S1 orthologs in gymnosperms, showing the conservation of this group in all seed plants. This is notably the first time a SIRT assay is performed for non-eudicot sequences, and our results might therefore provide clues for further research on the mechanism by which S1 5′uORFs regulate translation in these sequences.

We also showed that, while S1 orthologs likely appeared in the common ancestor of spermatophytes, the S subfamily as a whole is even older, dating back to charophytes. This finding completely abolishes the previous notion of S bZIPs as an angiosperm-specific group, and by making possible the study of ancient family members, could facilitate our understanding of modern S ortholog functions, possibly inherited and exploited through subfunctionalization.

### A model for the early evolution of the C/S1 bZIP transcription factor network

**Figure 4:**
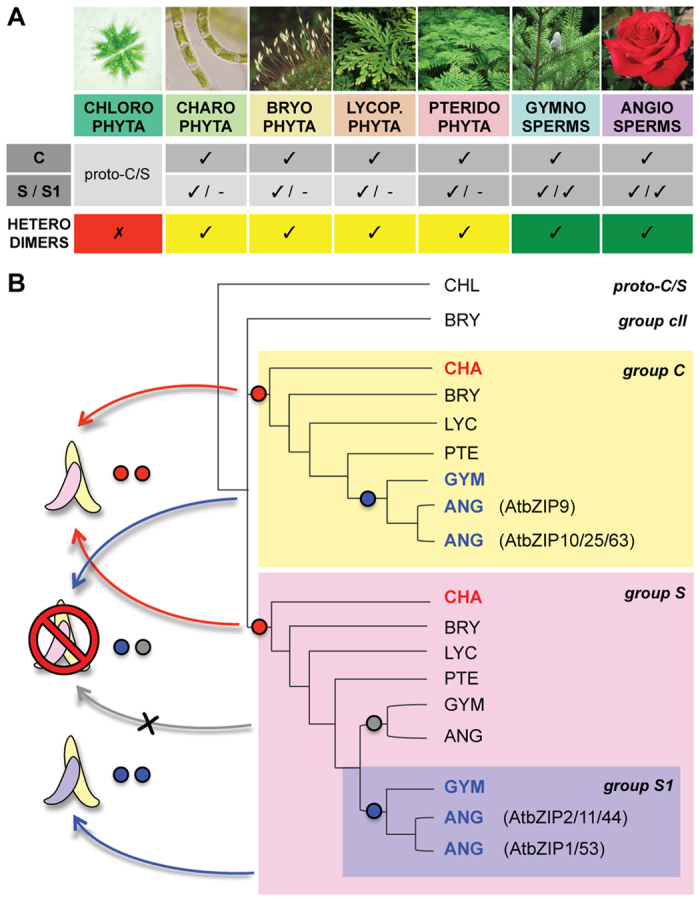
Presence pattern of C and S bZIP orthologs in different plant lineages and a model for the evolution of the C/S1 dimerization network. (**A**) Schematic summary of C and S or S1 bZIP orthologs coexistence in different plant lineages according to our results. Ticks and dashes indicate presence and absence, respectively. Based on the presence pattern, the putative formation of C/S or C/S1 heterodimers can be postulated (lowest row, yellow and green background respectively). (**B**) The gene tree summarizes the findings presented in this paper, and illustrates a possible model for the emergence of the C/S1 bZIP dimerization network as described in the text. Abbreviations refer to the plant lineage names used in panel A. Dot pairs and dimer cartoons provide information on approximate time of appearance and ortholog group of the bZIP sequences involved in hypothetical interactions, respectively. (Photographic references: the chlorophyta picture (https://commons.wikimedia.org/wiki/File:Micrasterias_.jpg) is public. The charophyta picture (https://commons.wikimedia.org/wiki/File:Klebsormidium_bilatum_Belgium_%2814759117646%29.jpg) is licensed under the Creative Commons Attribution 2.0 Generic license. The license terms can be found on the following link: https://creativecommons.org/licenses/by/2.0/deed.en. The bryophyta picture (https://commons.wikimedia.org/wiki/File:Bryi1004.JPG) is licensed under the Creative Commons Attribution-Share Alike 4.0 International license. The license terms can be found on the following link: https://creativecommons.org/licenses/by-sa/4.0/deed.en. The lycopodiophyta, pteridophyta, gymnosperms, and angiosperms pictures (https://commons.wikimedia.org/wiki/File:Selaginella_canaliculata.jpeg, https://commons.wikimedia.org/wiki/File:DidzialapisSakys.JPG, https://commons.wikimedia.org/wiki/File:Abies_homolepis_cones.jpg, https://commons.wikimedia.org/wiki/File:Rosa_Red_Chateau01.jpg, respectively) are licensed under the Creative Commons Attribution-Share Alike 3.0 Unported license. The license terms can be found on the following link: https://creativecommons.org/licenses/by-sa/3.0/deed.en).

Due to the long coexistence of C and S bZIPs before the appearance of S1 orthologs (Fig. 4A), we propose that S1 bZIPs may have inherited their specific heterodimerization preferences from ancestral S members; this seems more likely than S1 bZIPs abruptly acquiring dimerization capabilities with C bZIP partners, and vice versa. Ancestral C/S bZIP interactions in turn might have been retained from a homodimerizing proto-C/S bZIP ancestor, after the duplication event at the origin of *K. flaccidum* C and S sequences. We combined such observations into a consistent scenario for the emergence of C/S1 bZIP interactions, which we propose here (Fig. 4B). More importantly, our discovery of the first C subfamily representative also in charophytes uncovered the possible origin of C and S bZIPs as sister groups, duplicated from a common ancestral sequence. This hypothesis is again a novel insight, as the S bZIPs are currently proposed to have evolved from duplications within the C subfamily^2^.

To recapitulate, the steps leading to the C/S1 bZIP dimerization network emergence according to our model are proposed as follows: originally, an ancestral algal proto-C/S bZIP existed with homodimerizing properties. A later duplication of this sequence led to the generation of two paralogs, i.e. the ancestral C and S bZIP sequences observed in *K. flaccidum*, still able to interact. Such heterodimerization capability was maintained in the course of evolution, up to the S subfamily duplications in the common ancestor of spermatophytes, which created the conditions for the subfunctionalization of different S ortholog groups. Among them, S1 bZIPs specialized as C dimerization partners, while other S orthologs lost the heterodimerization capability. The latter step is the most hypothetical in our scenario, as the specificity of C/S1 interactions has been documented only in the model plant *A. thaliana*^13^. While it is likely that other eudicots present the same dimerization specificity, more distant species, such as monocots or gymnosperms, might still allow promiscuous C/S bZIP dimers. Concerning within-group dimerization capabilities, both C and S1 bZIPs showed varying interaction affinities, from nil to moderate, with themselves and other members of their ortholog group in *A. thaliana* protoplast two-hybrid experiments^13^; although comparably weaker than C-S1 interactions, heterodimerization seemed favored over homodimerization within each group. Experiments in rice also showed the capability of S1 member LIP19 to heterodimerize with OsOBF1, another S1 protein, but not with itself 53. It is however possible that other plant lineages evolved different within-group interaction preferences.

Independently from the exact steps correctly describing the C/S1 bZIP dimerization network emergence, comparing its timing with major events in plant evolution (e.g. speciation and colonization of new environments) would help clarifying its original role. For instance, the presence of C and S sequences in charophytes might have provided ancestral plants with an additional toolset for land colonization; later on, specialized C/S1 bZIP dimers in spermatophytes could have contributed to the complex adaptive features of both angiosperms and gymnosperms. While we showed the presence of both C and S bZIP sequences in basal species, the role of putative early C/S bZIP dimers remains a hypothesis.

### A reference for comparative studies on C and S1 bZIPs in non-model species

Our analyses clarified the details of C and S1 orthologs conservation in angiosperms, revealing lineage-specific duplications and gene losses in both subfamilies. Knowledge of such gain and loss patterns is necessary for the accurate transfer of functional information between model and crop species, and our results provide a more reliable classification framework than individual genome-specific bZIP catalogs published in recent years.

We believe that these findings will facilitate the study of the C/S1 bZIP transcription factor network in non-model species, suggesting new directions for experimental research on SIRT and heterodimers formation, and possibly leading to useful agricultural applications.

## Methods

### Sequences collection

Genome, transcriptome, and annotated protein data for several green plant species were collected from Phytozome v10^54^ and other online public repositories; included species, resources, and abbreviations are listed in Suppl. Table S1.

### Identification of C and S1 bZIP orthologs in angiosperms

Candidate angiosperm C and S1 bZIP transcription factors were retrieved from our protein or DNA sequence database using BLASTP v2.25+ 55 and HMMER v3.0^56^ searches, or TBLASTN v2.25+ 55 searches, respectively, with default settings. We used as queries annotated bZIP sequences (main ORF peptide sequence) from the arabidopsis TAIR10 release^57^ as dicot representative, from the rice TIGR release 7.0^58^ as monocot representative, and from the basal angiosperm amborella^46^; notice that rice and amborella sequences were used only in later search iterations, after phylogenetic assessment of their identity as C or S1 orthologs, and re-annotation of the peptide sequence for one of the amborella C bZIP sequences with the web version of AUGUSTUS (http://bioinf.uni-greifswald.de/augustus/)^59^. The previously annotated S subfamily OsbZIP79^2^ was excluded from the queries for two reasons: no match in our version of the rice genome, and divergent sequence features pointing to a pseudogene. Genomic hits were extended upstream and downstream by 1000 nucleotides and translated to amino acid sequence with the web version of AUGUSTUS (http://bioinf.uni-greifswald.de/augustus/)^59^; when this failed to produce satisfactory *de novo* predictions, guided protein predictions based on reference C and S1 bZIPs were generated using Exonerate v2.2.0 (https://www.ebi.ac.uk/~guy/exonerate/). The same tools were used to correct original protein annotations that appeared incomplete or mispredicted, after extracting their corresponding genomic region. Reverse BLASTP searches of the translated hits versus arabidopsis and rice proteomes (including labeled bZIP sequences) were used to remove obvious false positive hits, i.e. those without C or S bZIP sequences in the top 5 reverse matches. Given the high sequence similarity between S bZIP groups, we chose to include hits with best reverse matches to any S subfamily member, and not just to S1 bZIPs, to prevent the possible exclusion of relevant sequences. Reference arabidopsis and rice C and S bZIP sequences are shown in Supplementary Data S1.

### Identification of C and S bZIP orthologs in early branching species

The identification of C and S bZIP orthologs in chlorophytes, charophytes, bryophytes, lycopodiophytes, pterydophytes, and gymnosperms was performed as described above for angiosperms C and S1 bZIPs. Confirmed C and S orthologs from each species were iteratively used as queries to identify more distant sequences, potentially missed during the initial search with angiosperm queries.

### Phylogenetic analysis

C and S hits were aligned to the entire set of annotated arabidopsis and rice orthologs from the 13 known bZIP subfamilies, and phylogenetic reconstructions were performed to assess their identity as C or S subfamily orthologs. Confirmed candidates and reference arabidopsis and rice C or S bZIP were therefore re-aligned without members from other bZIP subfamilies to generate final high-resolution phylogenetic trees (not shown). For angiosperms S1 bZIP hits, a further phylogenetic reconstruction against reference S sequences from arabidopsis and rice was performed to better distinguish actual S1 candidates from other S hits (not shown). In addition to general C and S1 bZIP trees including all angiosperms species, lineage-specific alignments were generated independently to achieve a higher resolution of both C and S1 bZIP orthologs in different groups of flowering plants (Suppl. Figs S1 and S2). The trees obtained from these phylogenetic reconstructions were compared to the general C and S1 bZIP subfamilies trees, providing additional evidence for nodes with low bootstrap support values based on independent consistent topologies. C and S1 angiosperm sequences used in the building of phylogenetic trees are shown in Supplementary Data S1. For early branching species (gymnosperms and earlier), alignment were initially generated using only annotated protein sequences, and new translated genome hits were aligned to these in a later step (both version are shown in Suppl. Fig. S3); this strategy allowed us to build more reliable trees than by directly aligning a large number of newly predicted C and S bZIPs from genome, which would be strongly affected by an over-representation of gymnosperm sequences. Because of low bootstrap support values, multiple independent tree topologies for C, S, and C+S orthologs were again compared to infer consensus trees (Suppl. Fig. S3). C and S sequences from early branching species used in the building of phylogenetic trees are shown in Suppl. Data S1. Sequence alignments were performed using MAFFT v7.040, einsi algorithm^60^, and manually trimmed in Jalview v2.8.2^61^. Maximum likelihood phylogenetic reconstructions were performed with RAxML v7.2.8^62^ using 1000 bootstrap replicates, after selecting an appropriate amino acid substitution model with ProtTest v3.2^63^. The JTT+I+G model was used for all the trees shown in the Supplemental Information. Tree graphics was generated in iTOL v2.1^64^ and TreeGraph v2.4.0-456 beta^65^.

### Identification of 5′uORFs in S1 bZIP candidates

For each identified gymnosperms and amborella candidate S1 bZIP ortholog, the corresponding genomic region, including 1500 upstream nucleotides, was scanned with Exonerate v2.2.0 (https://www.ebi.ac.uk/~guy/exonerate/) for the presence of 5′uORFs similar to reference S1 sequences from arabidopsis and rice. The S1 bZIP ortholog from *Picea sitchensis* had to be excluded from the analysis, as the required upstream sequence was missing. Confirmed S1 5′uORFs were used as queries in a second search round to obtain additional hits, which allowed the identification of the truncated 5′uORF sequence from *Pinus abies.* Other gymnosperm S bZIP hits and more basal sequences from charophytes, bryophytes, lycopodiophytes, and pterydophytes were also analyzed to assess the presence of S1-like 5′uORFs. False positives were removed through manual inspection of sequence alignments, which were performed using MAFFT v7.040, linsi algorithm^60^. Identified 5′uORF sequences from gymnosperms are shown in Suppl. Data S1.

### Plant material and growth conditions

Plant material was obtained from arabidopsis Columbia-0 (Col-0) ecotype. Seeds were stratified in the dark at 4 °C for 2 days on soil, after which adult plants were grown at 22 °C under a 16 h light/8 h dark regime (100 *μ*mol m^−2^ s^−1^). Leaves of 4-week old plants were used in transient expression experiments.

### Construction of *P. abies* and *P. taeda* 5′-leader vectors

GeneArt^®^ gene synthesis service (Life Technologies, Carlsbad, CA, USA) was used to custom synthesize the 5′-leader sequences Arabidopsis S1 bZIP homologous genes from gymnosperm species *Picea abies* and *Pinus taeda*. Synthesized 5′-leader sequences contained the full 5′-leader, starting 500 nucleotides upstream of the arabidopsis *bZIP11* 5′uORF sequence. Gateway^®^ cloning sites attL1 and attL2 flanked the sequences, and the resulting constructs were cloned in the pMK-RQ vector backbone. Construct sequences are shown in the Supplementary Methods. Sequences were cloned into the pUC19 based p35S-ccdB-fLUC destination vector^24^, using Gateway^®^ LR cloning according to manufacturer instructions (Invitrogen, ThermoFisher Scientific, Waltham, USA) to create transient *LUC* expression vectors.

### Transient transformation of arabidopsis material

Transient transformation of arabidopsis seedlings was performed as previously described^24^, with minimal adjustments. DNA coating of gold particles was performed accordingly, using 1.2 mg of fLUC vector and 0.4 mg of rLUC normalizing vector per transient expression experiment. Plant material was transformed using the Biolistic particle delivery system, model PDS-1000 He (Bio-Rad, Hercules, CA, USA). Leaves from 4 weeks old plants were transformed using 900 psi rupture discs. Two leaves were simultaneously transformed, after which one was incubated in 10 mL liquid one-half strength MS-medium supplemented with 6% sorbitol, and the other in medium containing 6% sucrose. Incubations were performed in 100 mL flasks, which were placed on a rotary shaker (50 rpm) under constant light for 24 hours. Material was harvested, washed with demi-water and frozen in liquid nitrogen. Samples were stored at −80 °C.

### LUC activity assays

Protein extracts from transformed leaf material expressing *LUC* were made from approximately 25 mg ground tissue using 100 μl of Cell Culture Lysis (CCL) reagent (Promega, Madison, WI, USA, #E1531). Plant powder was incubated in extraction buffer for 10 minutes at room temperature, followed by 5 minutes of centrifugation (16,000 xg). Twenty microliters of supernatant was transferred to a white 96 well luminometer plate (Promega, #Z3291). LUC activity was measured with a Glomax 96 microplate luminometer (Promega), using the “LUC assay system with injector” protocol of the Glomax software. Relative LUC-levels of transiently transformed plant material was determined by the ratio of fLUC to rLUC activity, as previously described^24^. 100 μL of the substrates supplied in the Dual Luciferase assay kit (#E1960, Promega), was applied to measure fLUC and rLUC activity. LUC activity was assayed with a 10 second integration time and a 2 second delay between injection and measurement.

## Additional Information

**How to cite this article**: Peviani, A. *et al.* The phylogeny of C/S1 bZIP transcription factors reveals a shared algal ancestry and the pre-angiosperm translational regulation of S1 transcripts. *Sci. Rep.* **6**, 30444; doi: 10.1038/srep30444 (2016).

## Supplementary Material

Supplementary Information

Supplementary Table S1

Supplementary Table S2

Supplementary Dataset 1
