# Supplementary material for: The phylogeny of C/S1 bZIP transcription factors reveals a shared algal ancestry and the pre-angiosperm translational regulation of S1 transcripts
Source: Sci Rep. 2016 Jul 26;6:30444. doi: 10.1038/srep30444 (PMC4960570; doi:10.1038/srep30444)

"The phylogeny of C/S1 bZIP transcription factors reveals a shared algal ancestry and the pre-angiosperm translational regulation of S1 transcripts". Peviani A., Lastdrager J., Hanson J., Snel B.

Supplementary Methods: Gymnosperms constructs used for the SIRT experiments. Custom synthesized nucleotide sequences of *P.abies* and *P. taeda* S1-like gene leaders, flanked by Gateway® cloning sites attL1 and attL2 (red). The AtbZIP11 5'ORF homologous section is indicated in bold.

P. abies

CACTATAGGGCGAATTGAAGGAAGGCCGTCAAGGCCGCATCAAATAATGATTTTATTTTGACTGATAGTGACCTGTTCG
TTGCAACAAATTGATGAGCAATGCTTTTTTATAATGCCAACTTTGTACAAAAAAGCAGGCTCGTCCAATACTTGCTTCC
CAATTCTGGGCTTTAGCCAGAAGCGGACTTGCTTGCATTCCTGTATTGGGTGTATAATTTTAACTCTTTAATTTTTGTT
TTCTTTTTCAAAAAATTGATACTGGATGCAATTGTGATCCTGCAGAGCCATTCCTAAATCAAATAGCCTTCTCTAAATC
AAATAACCTTCTCTGCCTGCAAATAAGCAAGTAGGGCAAGTGCATTAGATCTAGATCCTACATCTAGCTTTTCACAATC
TTTTCTCAAGGCAAAGGCAATTGTGACGCTGCAGAGCTATTGCTAAATCGAAATAGCCTTCTCCCCGCCCAAGCACAAG
TAGGGCCAGTGCATGAGATCTAGATCCTGCATCTAGATTTTCACAATCTTTCGTTAAGGCACTTAGATGCAATTAGATG
AACTAAAAACTAAATTGCTGTAAAGTCCATAATCAAGAATTTATATTATTGGTACCTAAGATTGCACTCTTCTTGATAT
TGGATTTT**ATGTTTCCATCCTCACTACTGGTTTCTGGGGTTCTACTCATAGTTTTTCAACTATTTTTGTATTGGATTTA**
CAGATTCATGTCCAATGATTACTGCCCATATCTGAAAATTCTCCTCTGTTTGCATTAGTGCGTTGGATTTCTGTTTTTT
TACCTCTATTACTCATCGCAATCACTGCCTAATTCTGTGGTTTTTGCAGCAAGCCTGACATATAAGCTTTGCAGTAGGC
ACCCAGCTTTCTTGTACAAAGTTGGCATTATAAGAAAGCATTGCTTATCAATTTGTTGCAACGAACAGGTCACTATCAG
TCAAAATAAAATCATTATTTGCTGGGCCTCATGGGCCTTCCTTTCACTGCCCGCTTTCCAG

P. taeda

CACTATAGGGCGAATTGAAGGAAGGCCGTCAAGGCCGCATCAAATAATGATTTTATTTTGACTGATAGTGACCTGTTCG
TTGCAACAAATTGATGAGCAATGCTTTTTTATAATGCCAACTTTGTACAAAAAAGCAGGCTCTGATATTGGATGCACTT
GTGATCCTGTAGGGTCGTTACAAAATCAAGGAGCCTTCTCTGAATCAAATAGCCTTCTCTCCTGCAAATACCAAATAAG
CAAGTAGAGCAAGTGCATCAGATCTACATCCTACATCTAGTTCCCCGCATTCTTTTCTAAAGGCAAATGCGATTTTGAT
GCTGCAGAGCTATTGCTAAATCGAAATAGCTATCTCCCTTCACATAAGCAGCTAGGGCAATTGCATCAGATCTAGATCC
TTTCTAAGGGAAAATGCAATTGTGACGTTGCAGAGCTTTTGCTAAATCGAATTAGCCTCCTCCCCGCCACAAGCGCAAG
CAGGACCAGTGCATGAGATCTAAATCCTACATCTAGATTTGCACAATCTTTCGTTAAGACGCTTAGATGCAATCAGCTG
AACTAGAAAGTAAACTGTTATAAAGTCCATAATCAGGAATTTATATTACTGTTATCTAAGATTGCACTCTTCTTGACTT
TGGATTTT**ATGTTTCCATCCTCGCCACTGGTTTCTGGGGTTCTACTTATAGTTTTTCACCTATTTTTGTATTGGATTTA**
TAGATTCATATCCCATAACTGAAAATTCTCCTCTGCTGCATTAGCGTGTTGGATTTCTGGTATTTACCTCTATTACTCA
TAGCAATCACTGCCTATCTCTGTGGTTTTTGCAGCAAGCCTCACAAATAAGCTTTGCAGTAGGCACCCAGCTTTCTTGT
ACAAAGTTGGCATTATAAGAAAGCATTGCTTATCAATTTGTTGCAACGAACAGGTCACTATCAGTCAAAATAAAATCAT
TATTTGCTGGGCCTCATGGGCCTTCCTTTCACTGCCCGCTTTCCAG

"The phylogeny of C/S1 bZIP transcription factors reveals a shared algal ancestry and the pre-angiosperm translational regulation of S1 transcripts". Peviani A., Lastdrager J., Hanson J., Snel B.

Figure S1: Phylogenetic trees of angiosperms C bZIPs.

Tree 1. C bZIP orthologs, all angiosperm species.

Tree 2. C bZIP orthologs, reference sequences from *A. thaliana* + brassicales species.

Tree 3. C bZIP orthologs, reference sequences from *O. sativa* + poales species.

Tree 4. AtbZIP9 orthologs.

Tree 5. AtbZIP10/25/63 orthologs.

Tree 6. AtbZIP10/25/63 orthologs, eudicot species detail.

Tree 7. AtbZIP10/25/63 orthologs, monocot species detail.

Tree 1. C bZIP orthologs, all angiosperm species

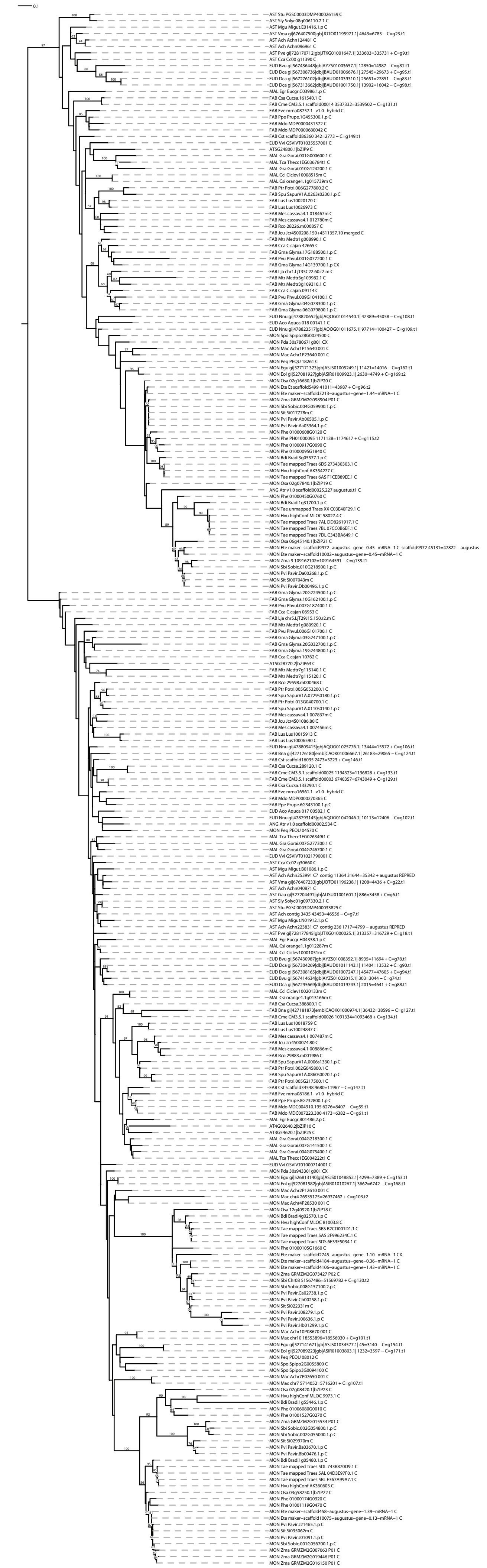

Tree 2. C bZIP orthologs, reference sequences from *A. thaliana* + brassicales species

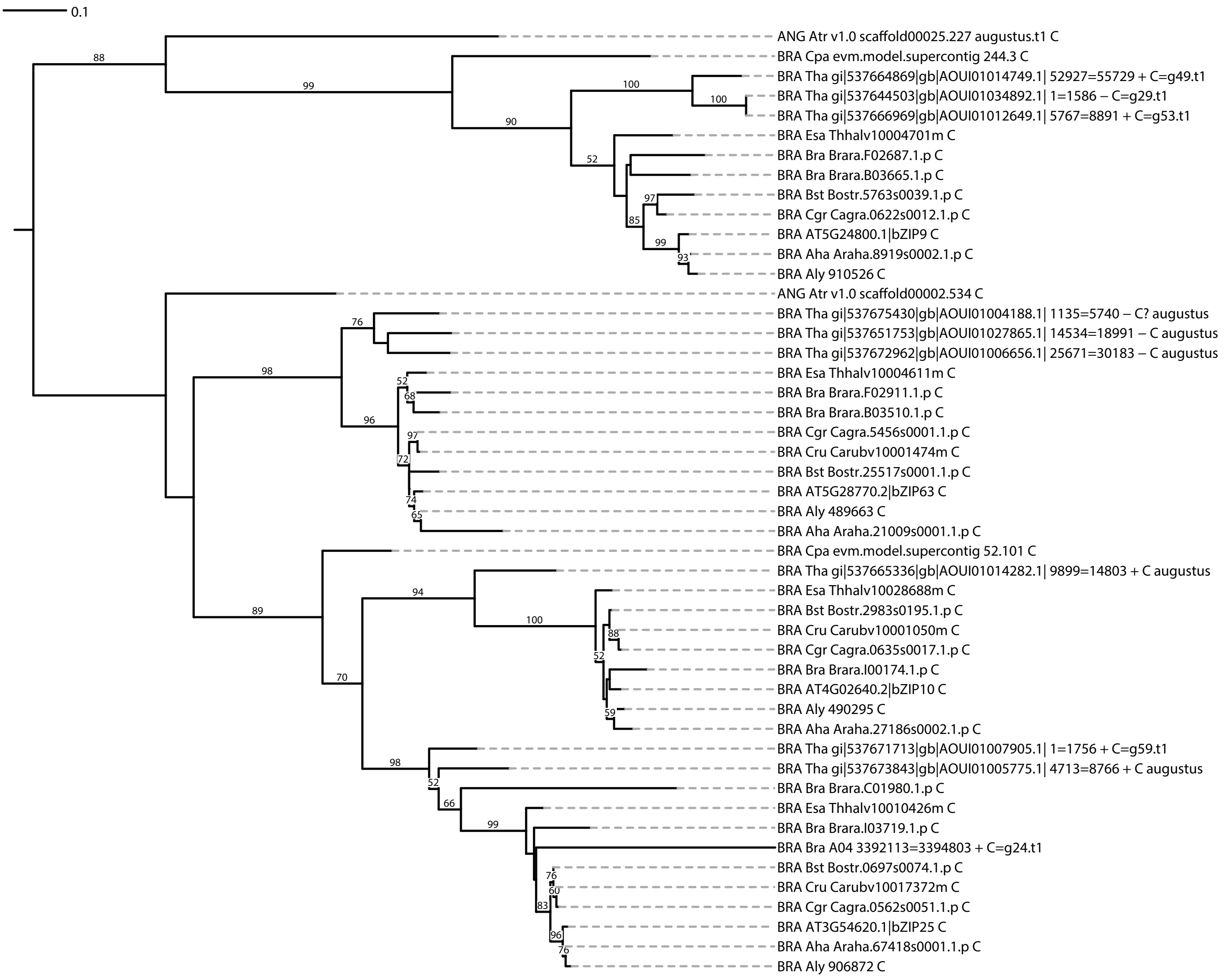

Tree 3. C bZIP orthologs, reference sequences from *O. sativa* + poales species

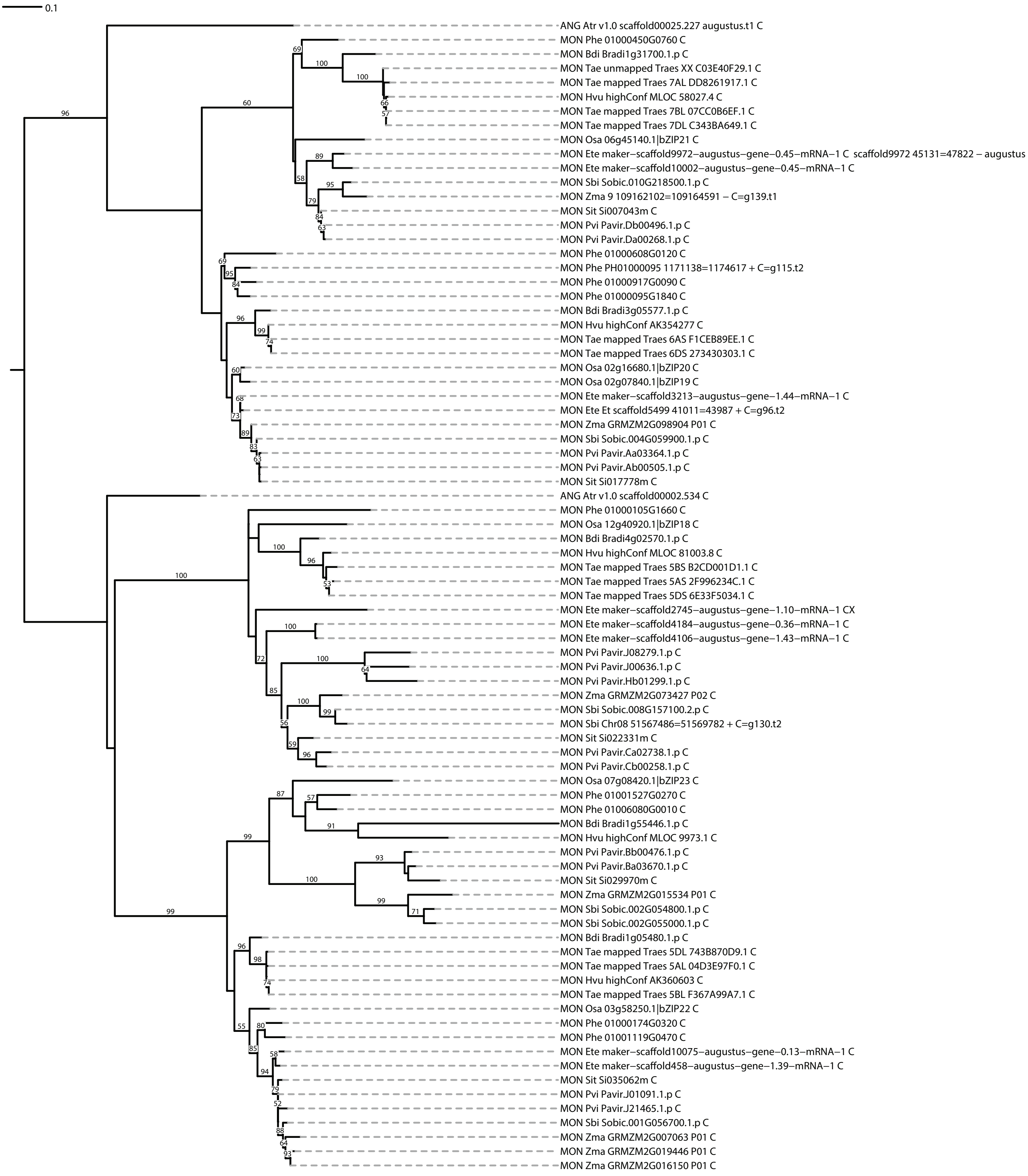

Tree 4. AtbZIP9 orthologs

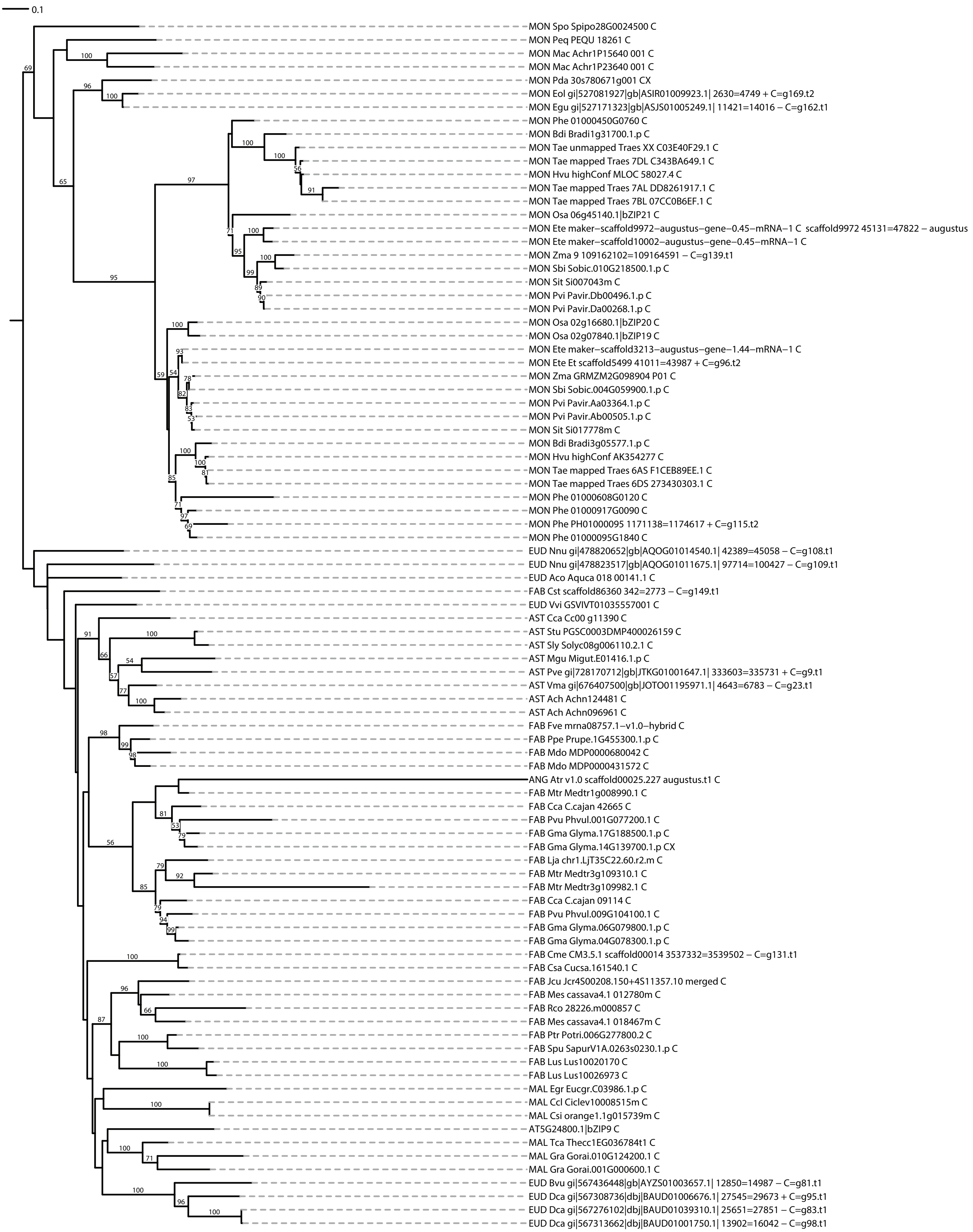

Tree 5. AtbZIP10/25/63 orthologs

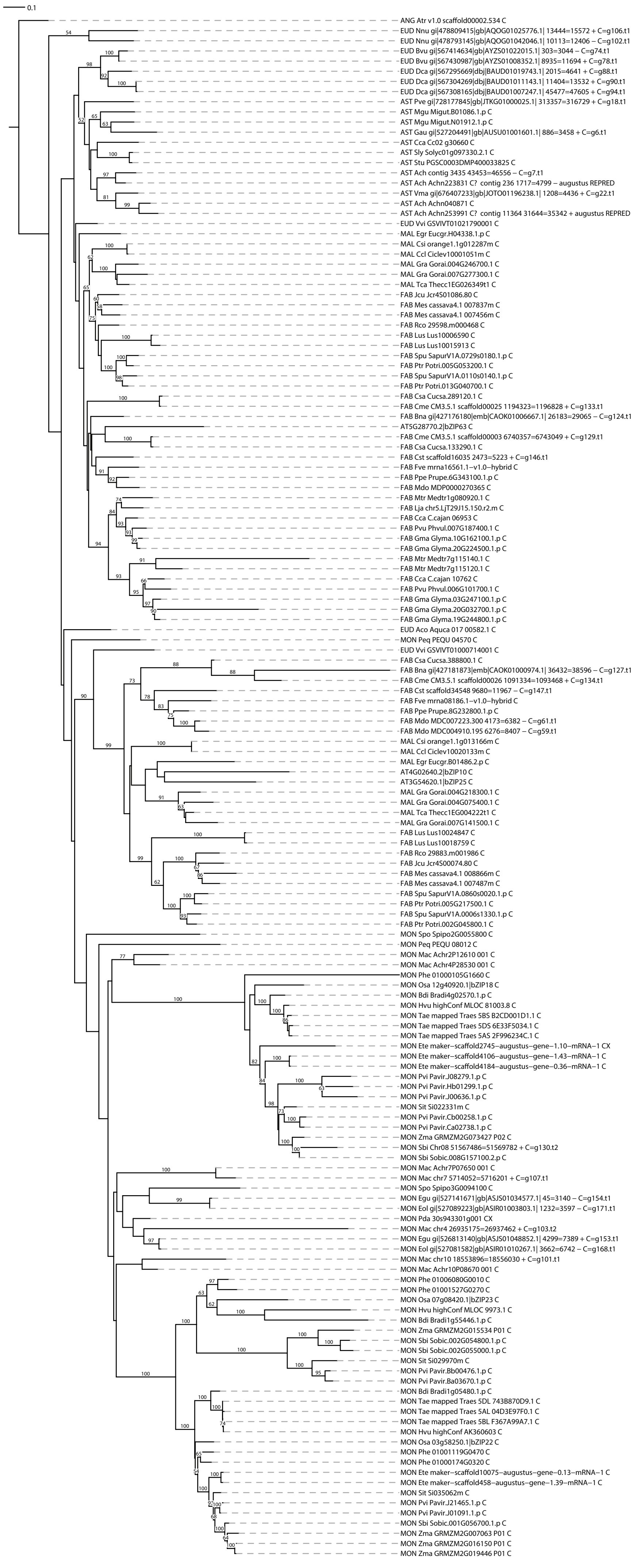

Tree 6. AtbZIP10/25/63 orthologs, eudicot species detail

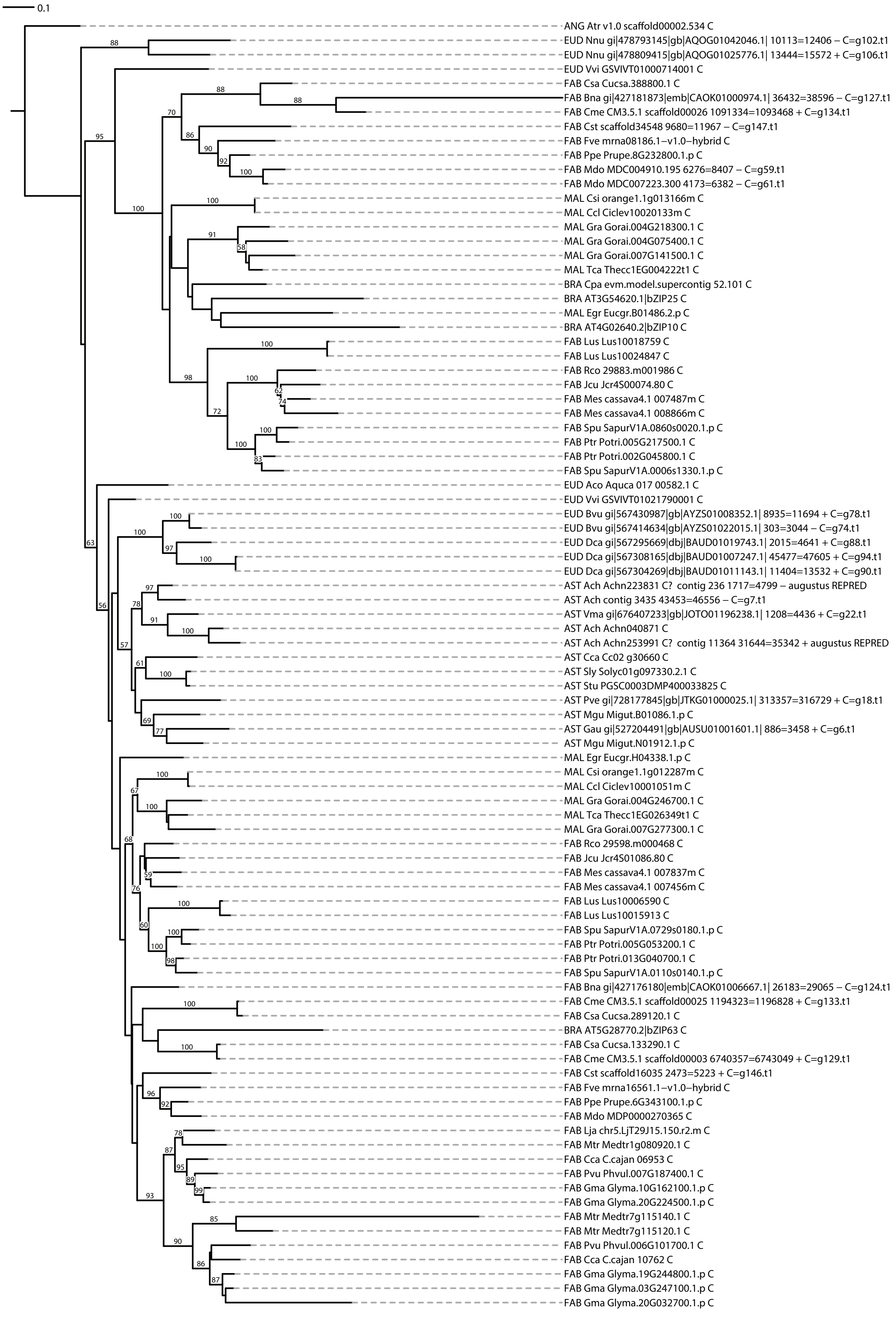

Tree 7. AtbZIP10/25/63 orthologs, monocot species detail

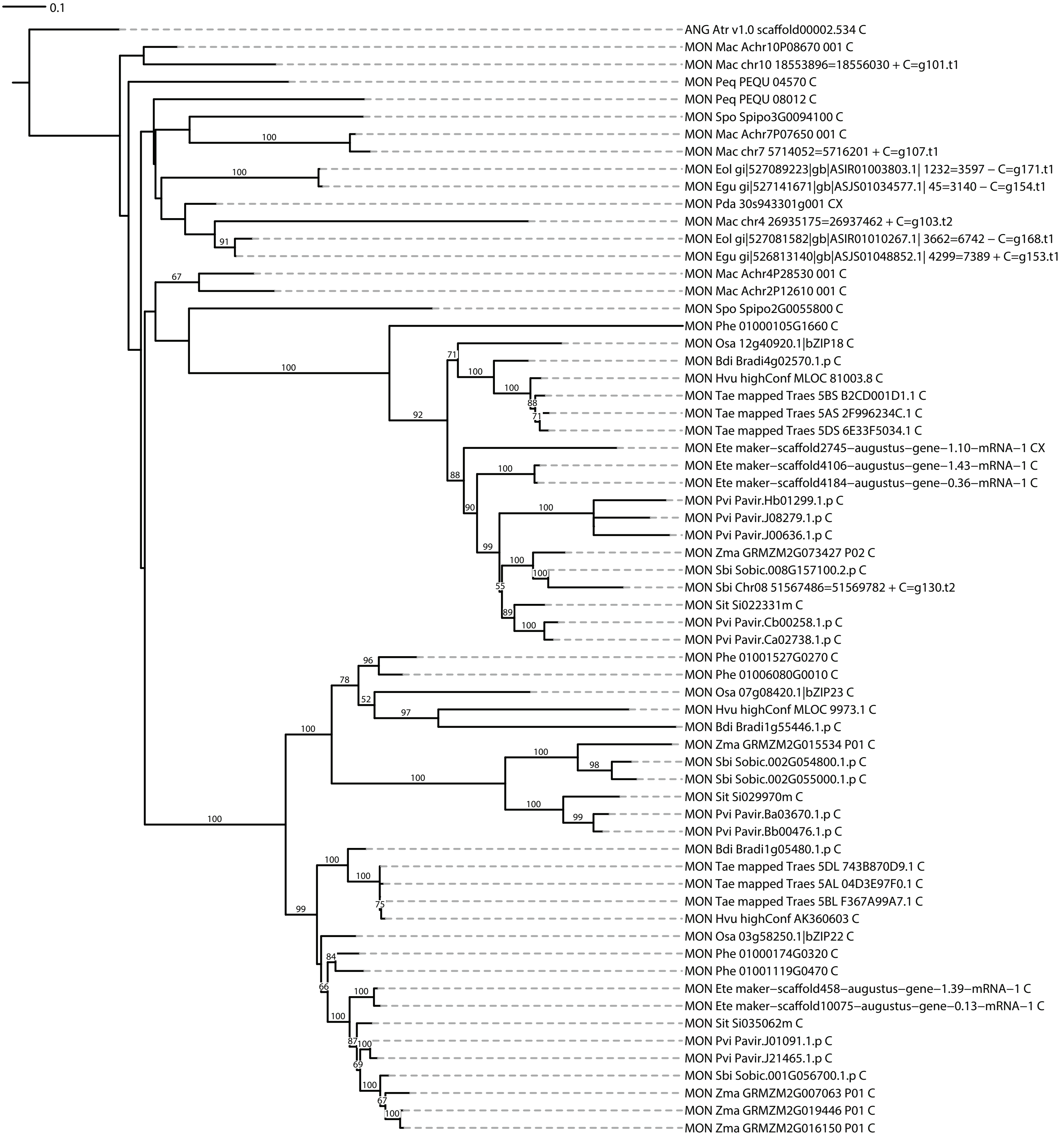

"The phylogeny of C/S1 bZIP transcription factors reveals a shared algal ancestry and the pre-angiosperm translational regulation of S1 transcripts". Peviani A., Lastdrager J., Hanson J., Snel B.

Figure S2: Phylogenetic trees of angiosperm S1 bZIPs.

Tree 1. S1 bZIP orthologs, all angiosperm species.

Tree 2. S1 bZIP orthologs, reference *A. thaliana* sequences + brassicales species.

Tree 3. S1 bZIP orthologs, reference *O. sativa* sequences + poales species.

Tree 4. S1 bZIP orthologs, basal eudicot + malvid species.

Tree 5. S1 bZIP orthologs, fabid species.

Tree 6. S1 bZIP orthologs, asterid species.

Tree 7. S1 bZIP orthologs, non-poales monocot species.

Tree 8. AtbZIP1/53 orthologs.

Tree 9. AtbZIP2/11/44 orthologs.

Tree 10. AtbZIP1/53 orthologs, eudicot species detail.

Tree 11. AtbZIP1/53 orthologs, monocot species detail.

Tree 12. AtbZIP2/11/44 orthologs, eudicot species detail.

Tree 13. AtbZIP2/11/44 orthologs, monocot species detail.

Tree 1. S1 bZIP orthologs, all angiosperm species

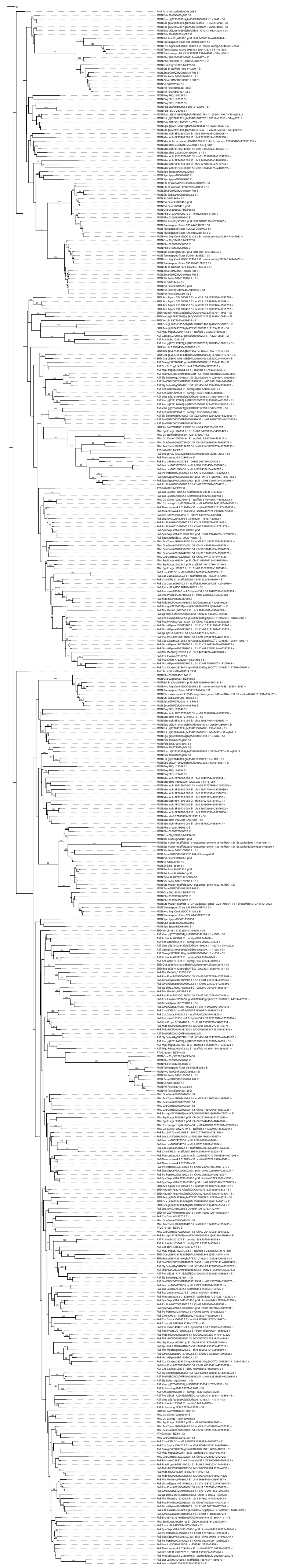

Tree 2. S1 bZIP orthologs, reference *A. thaliana* sequences + brassicales species

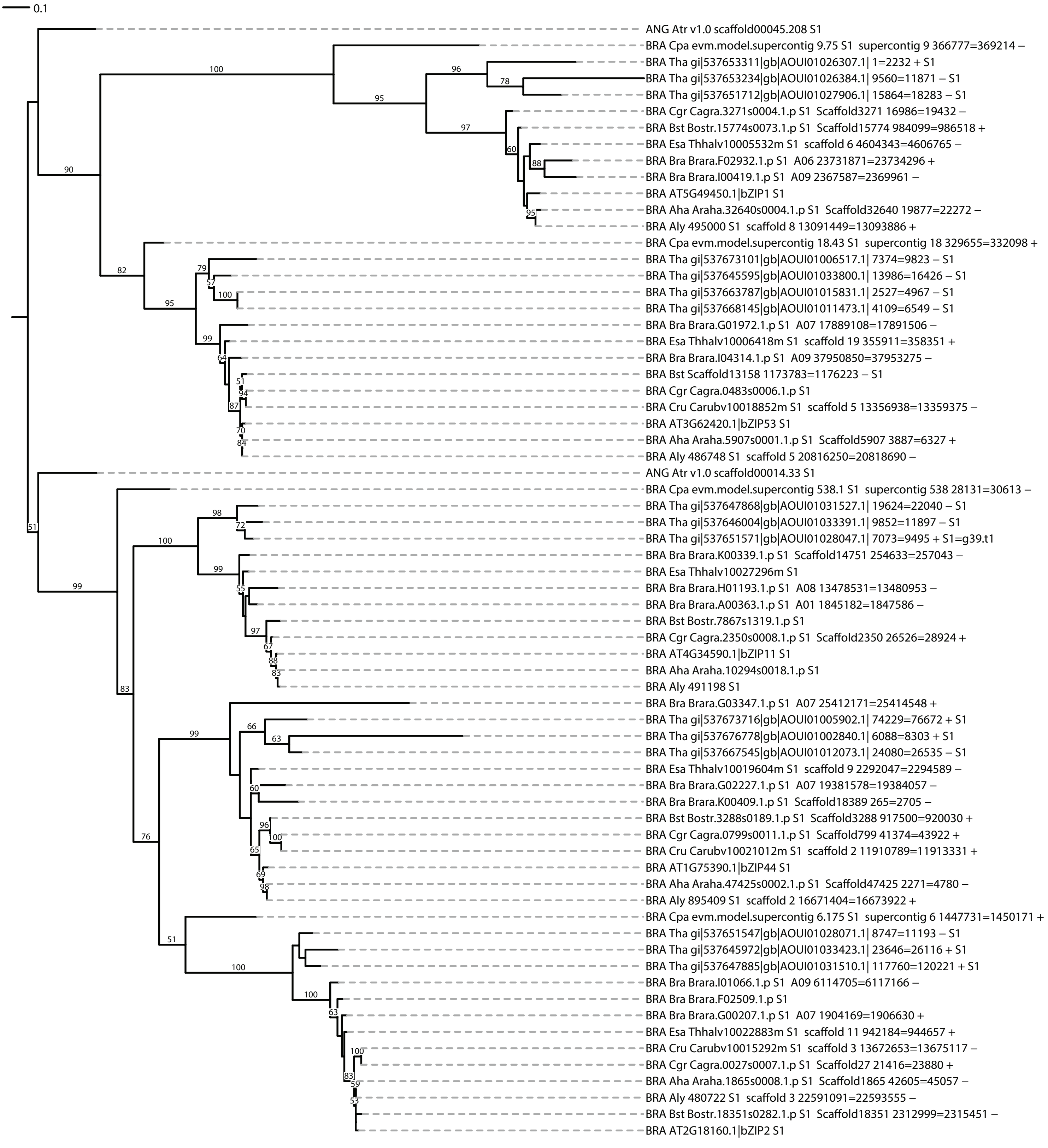

Tree 3. S1 bZIP orthologs, reference *O. sativa* sequences + poales species

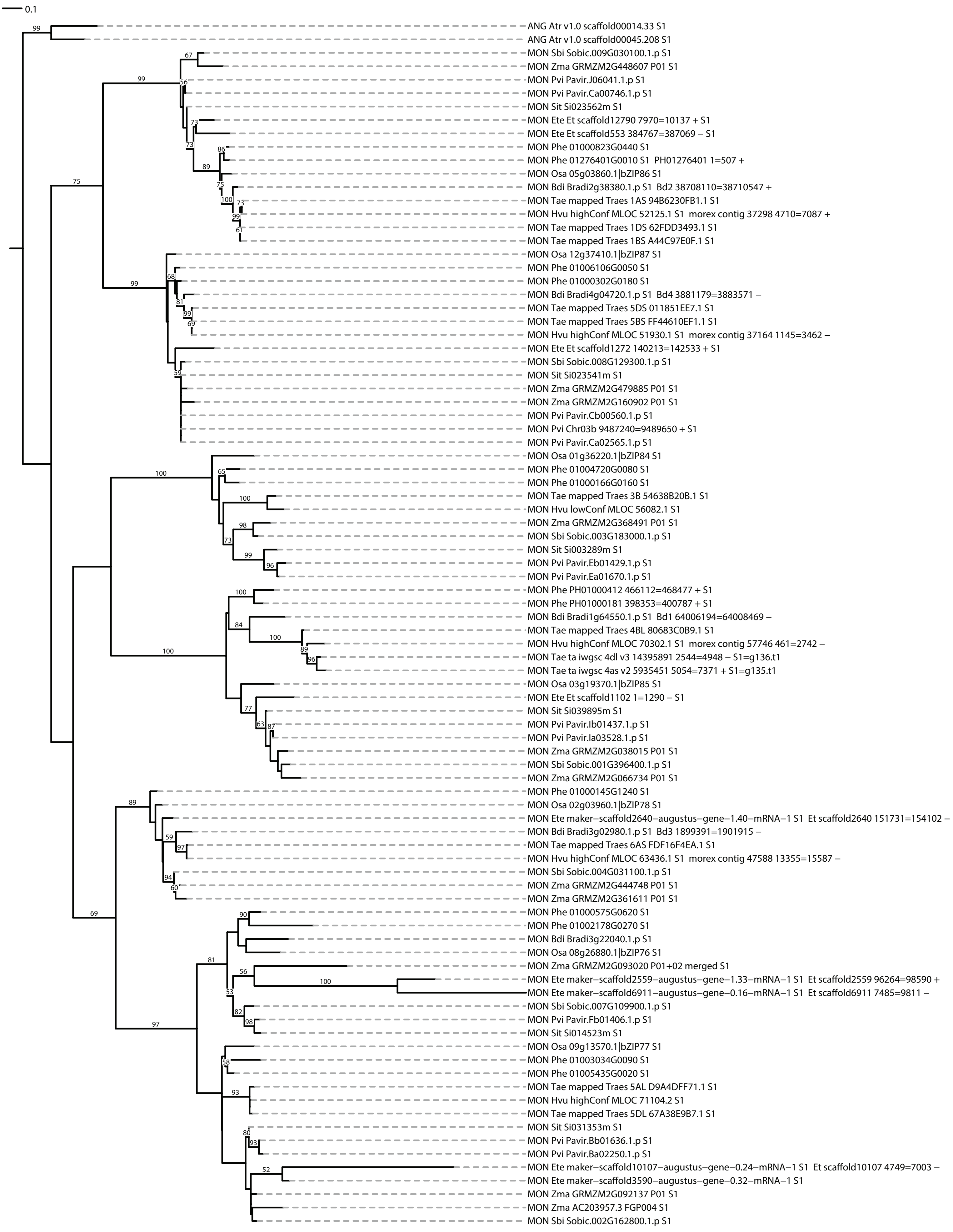

Tree 4. S1 bZIP orthologs, basal eudicot + malvids species

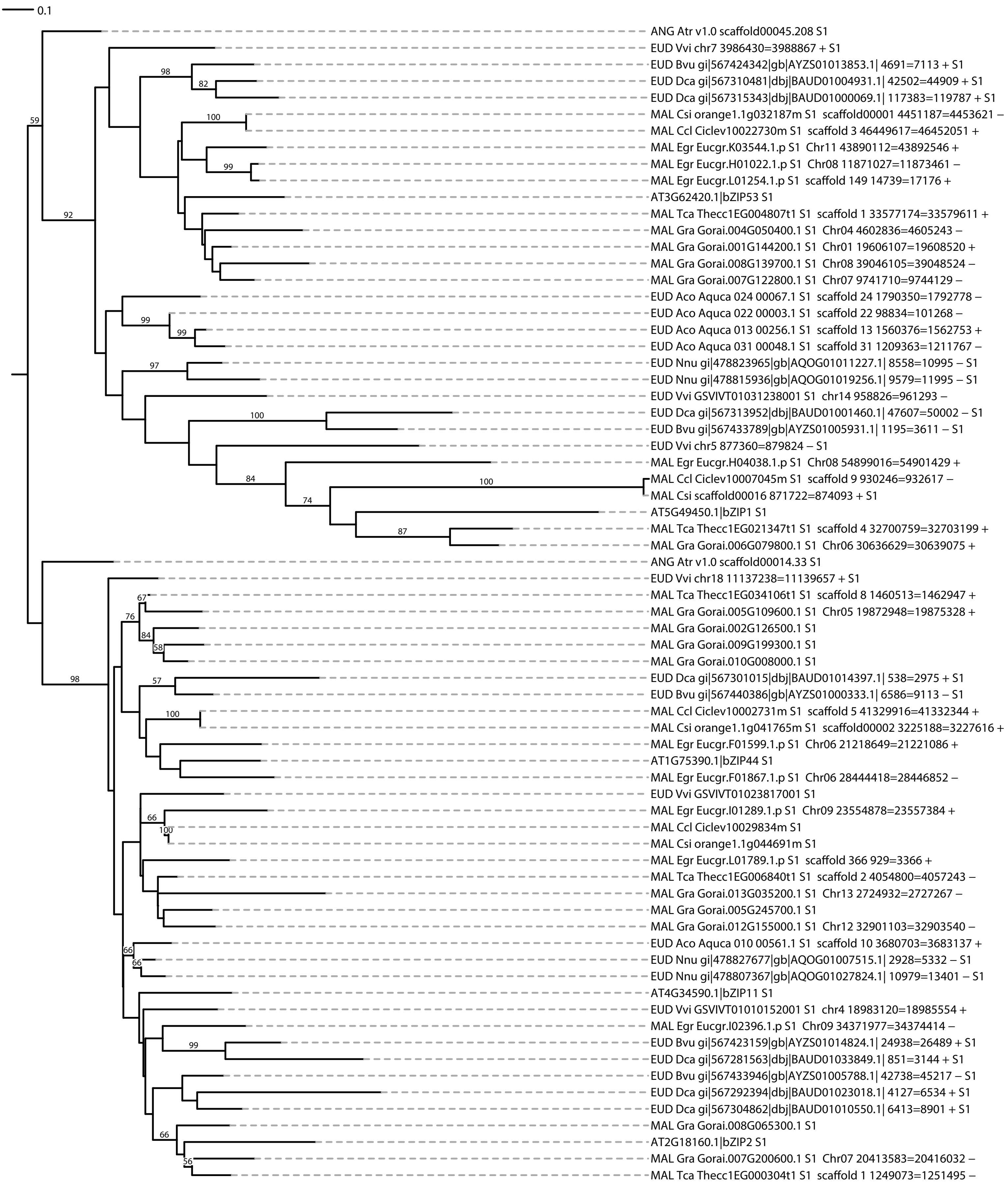

Tree 5. S1 bZIP orthologs, fabids species

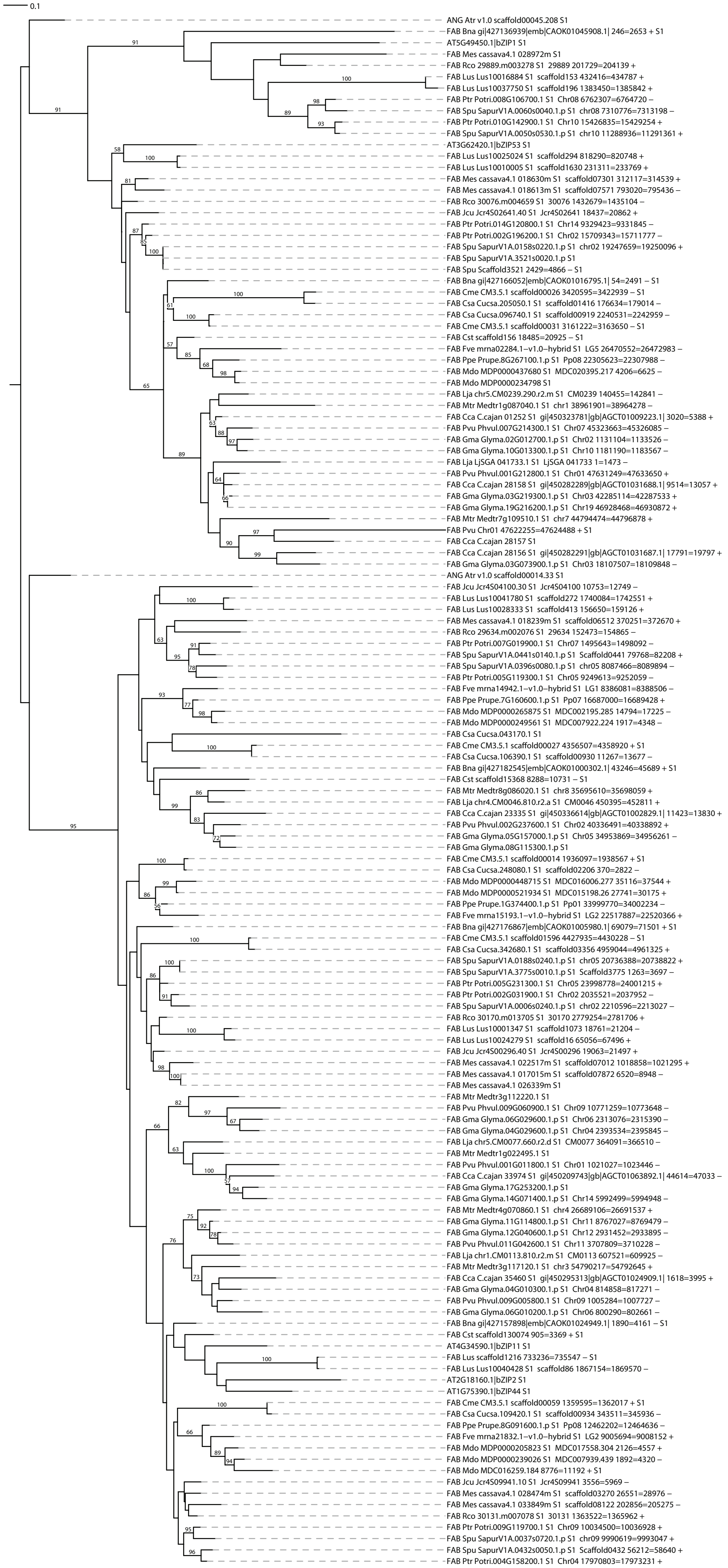

Tree 6. S1 bZIP orthologs, asterids species

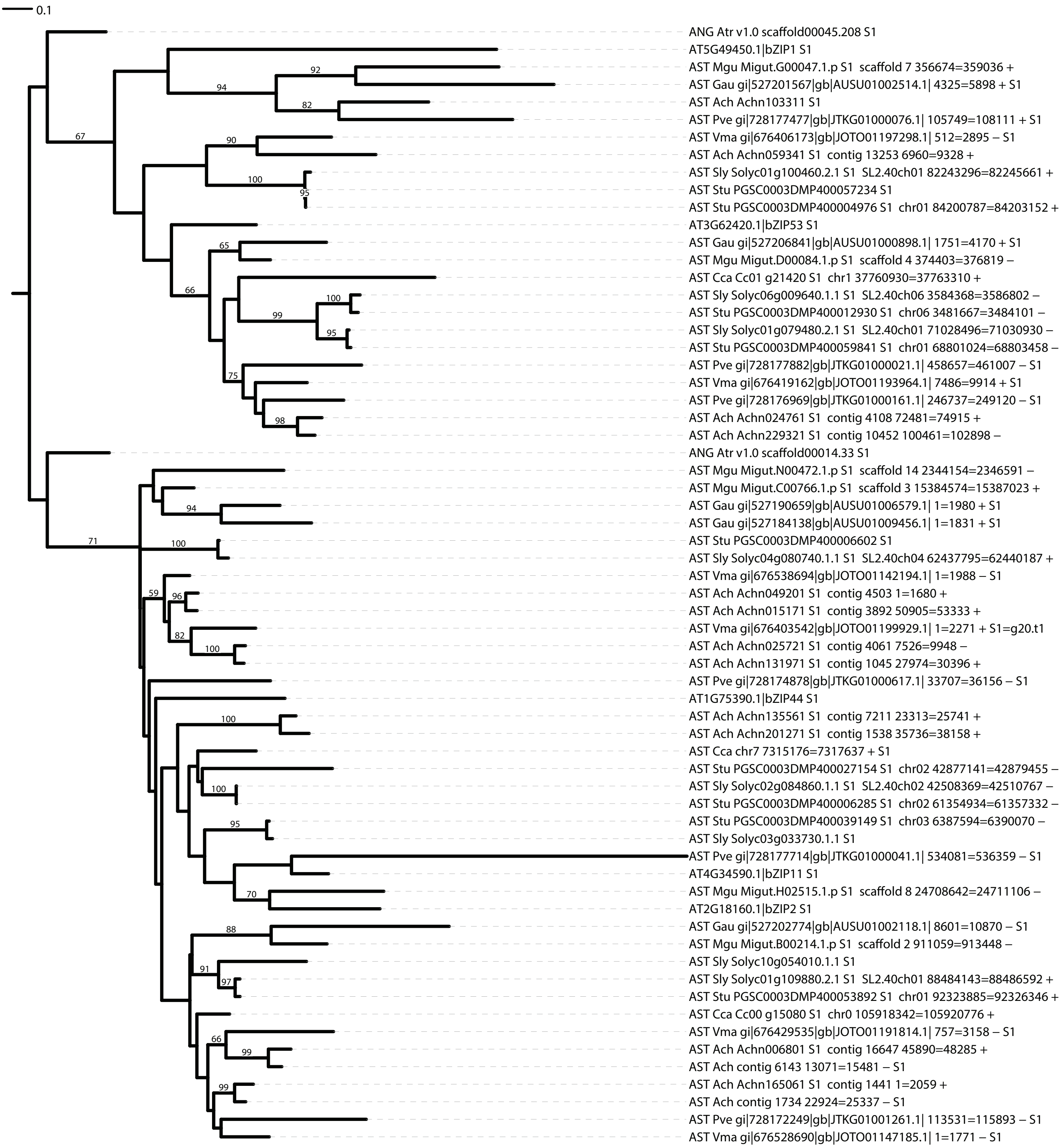

Tree 7. S1 bZIP orthologs, non-poales monocot species

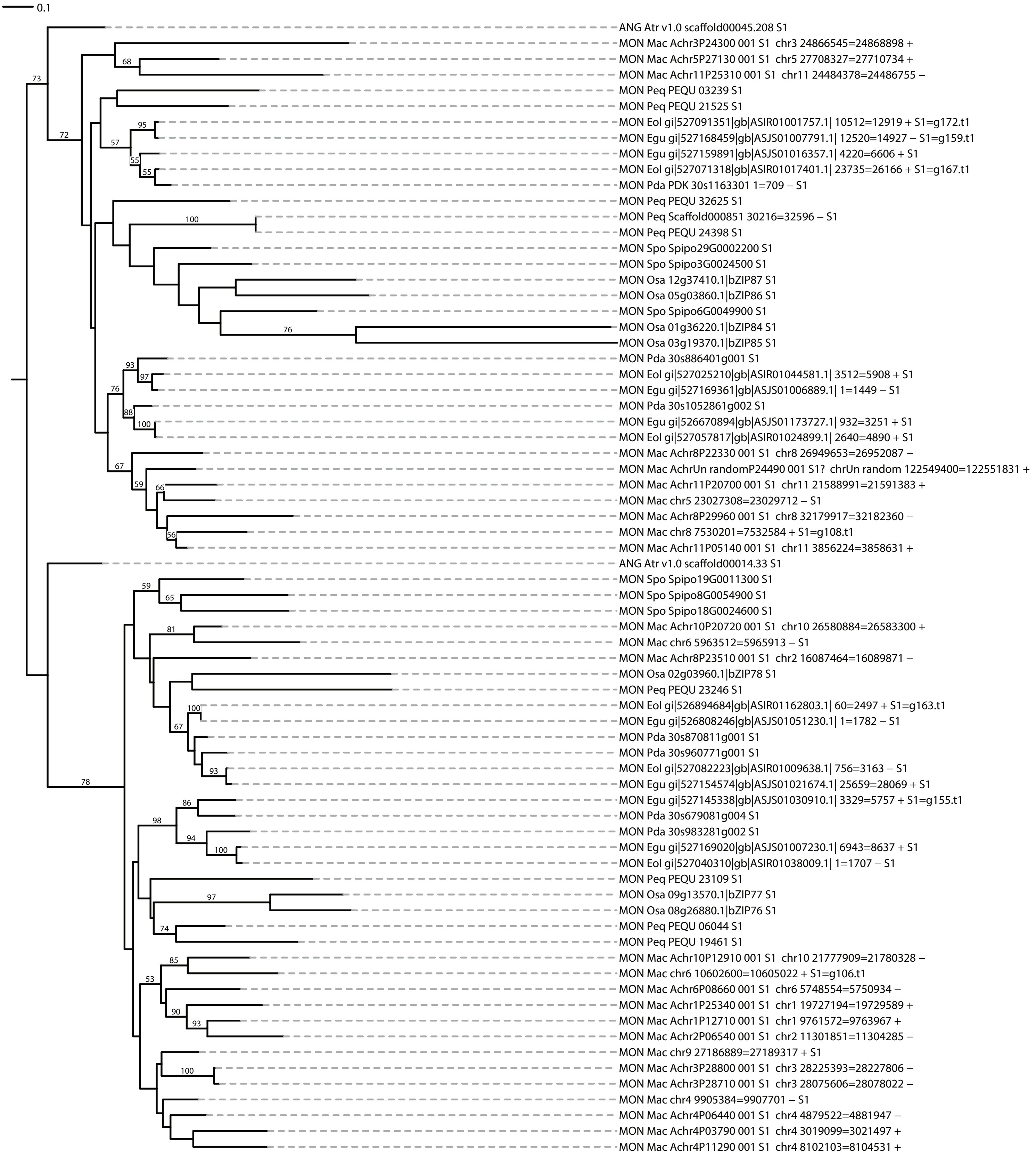

Tree 8. AtbZIP1/53 orthologs

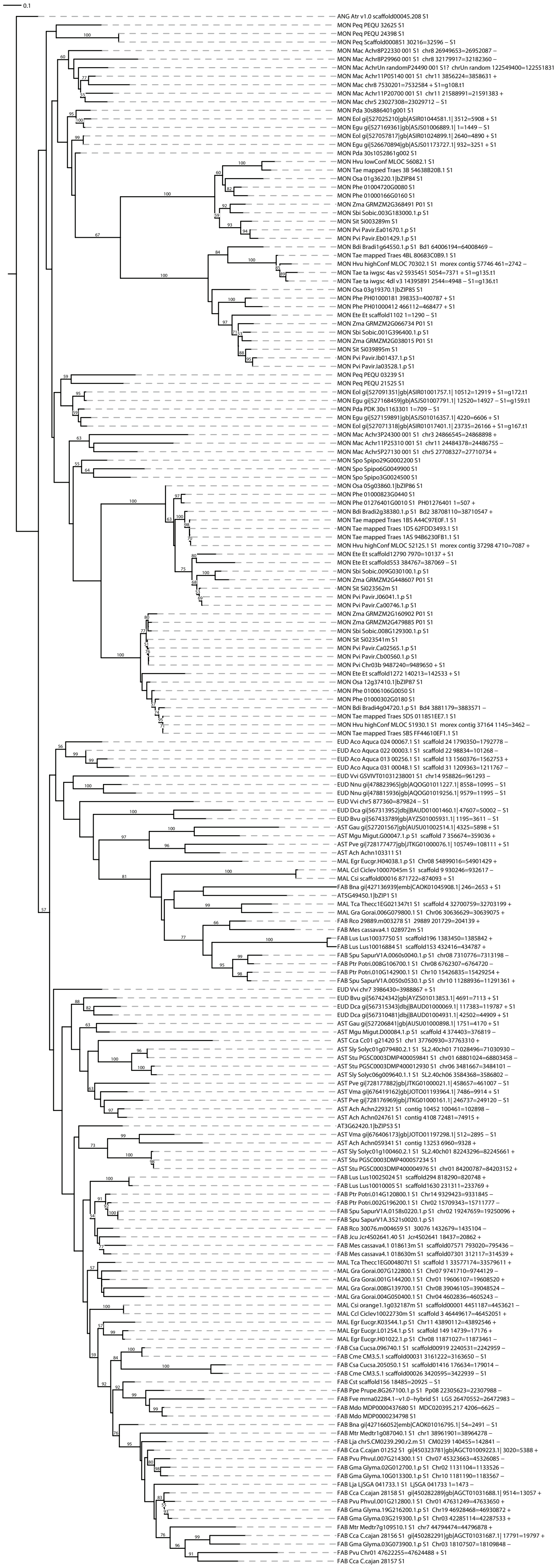

Tree 9. AtbZIP2/11/44 orthologs

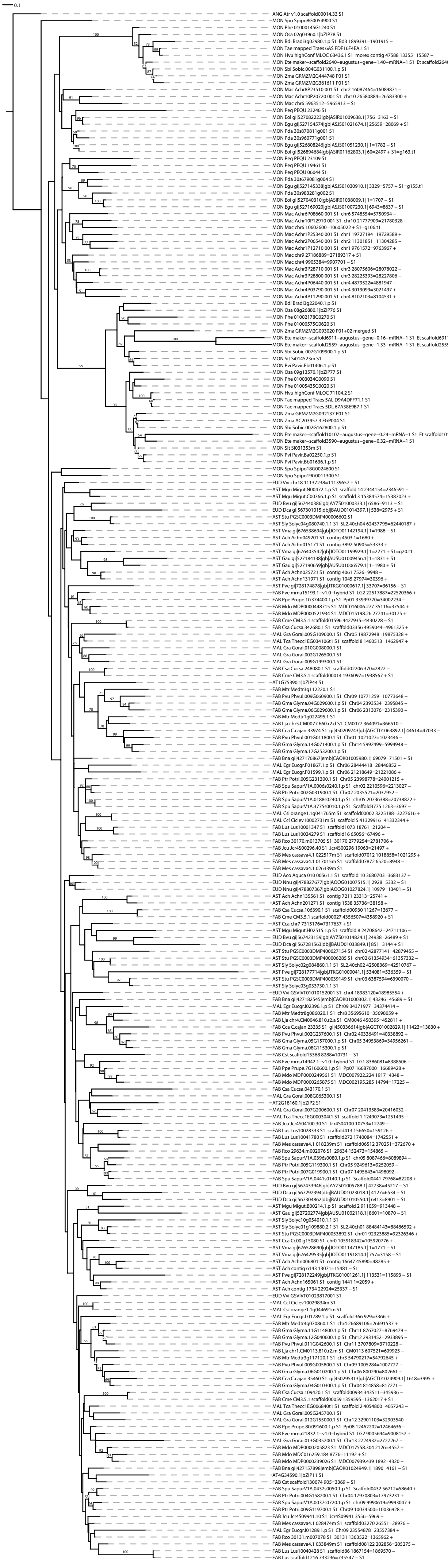

Tree 10. AtbZIP1/53 orthologs, eudicot species detail

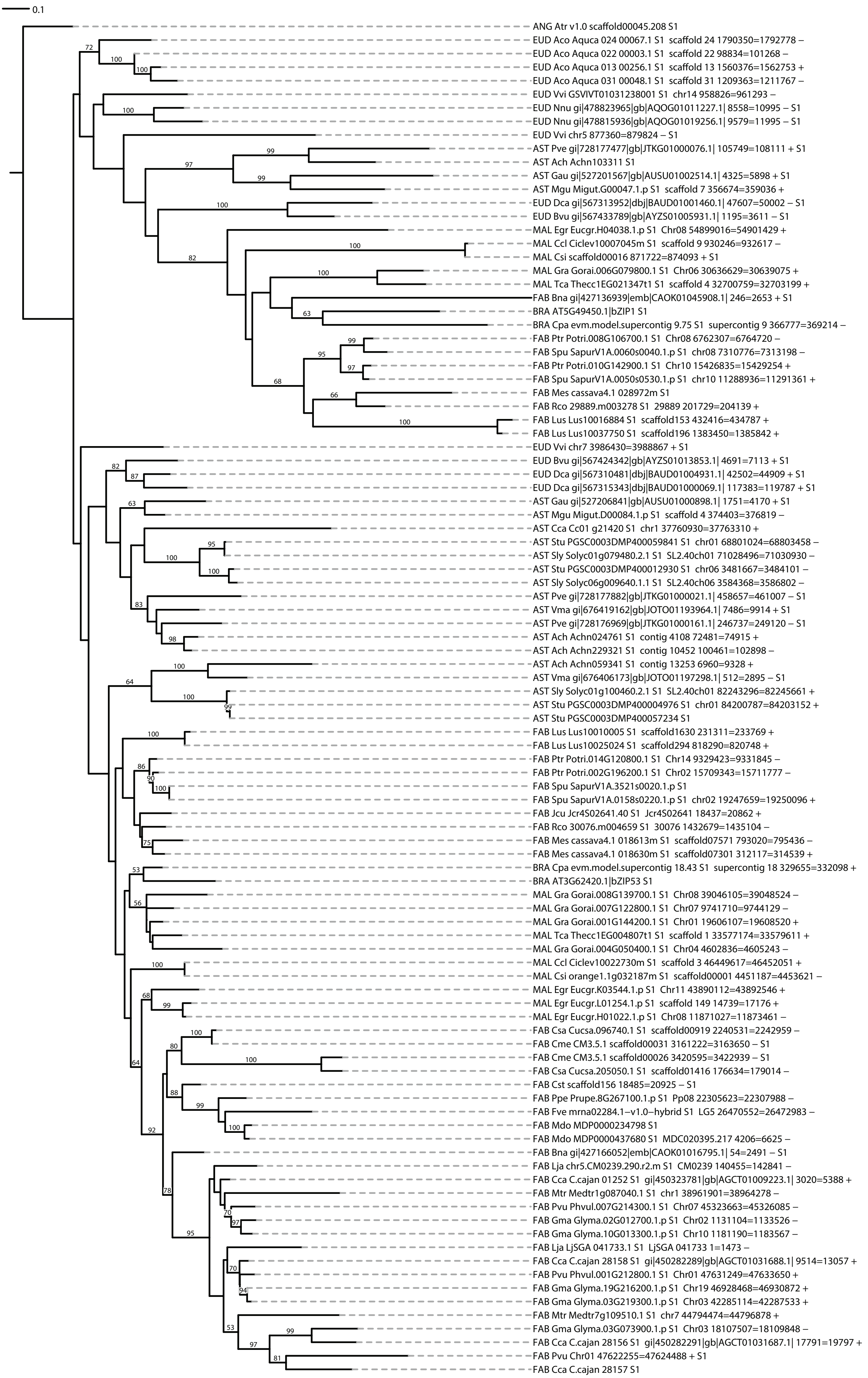

Tree 11. AtbZIP1/53 orthologs, monocot species detail

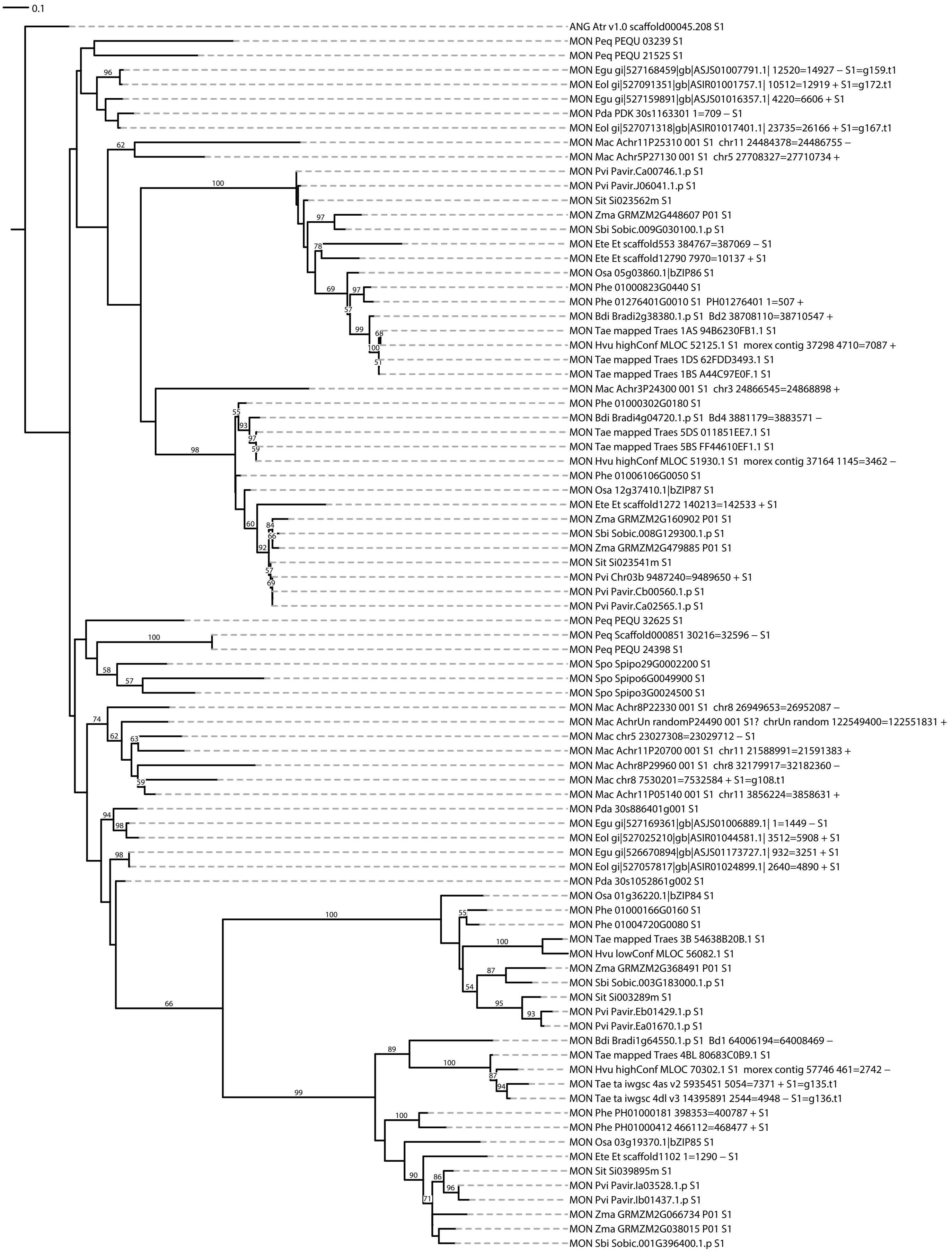

Tree 12. AtbZIP2/11/44 orthologs, eudicot species detail

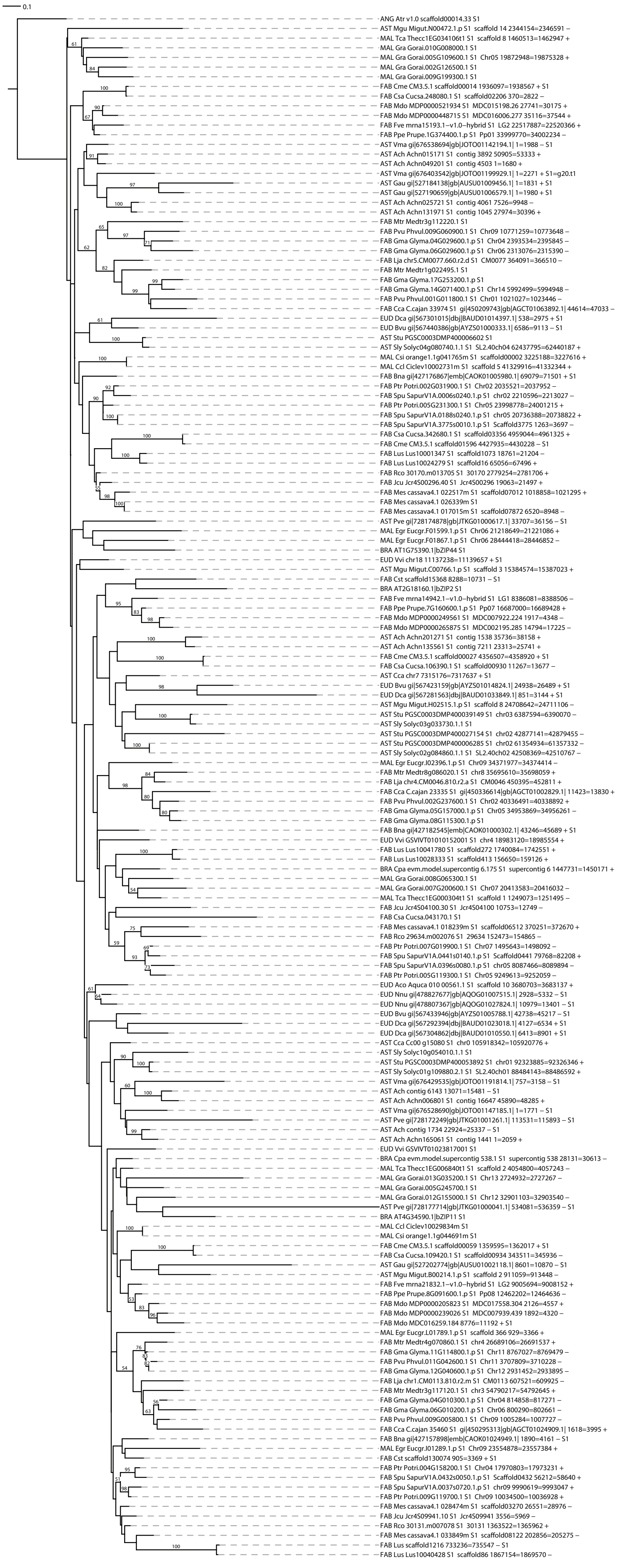

Tree 13. AtbZIP2/11/44 orthologs, monocot species detail

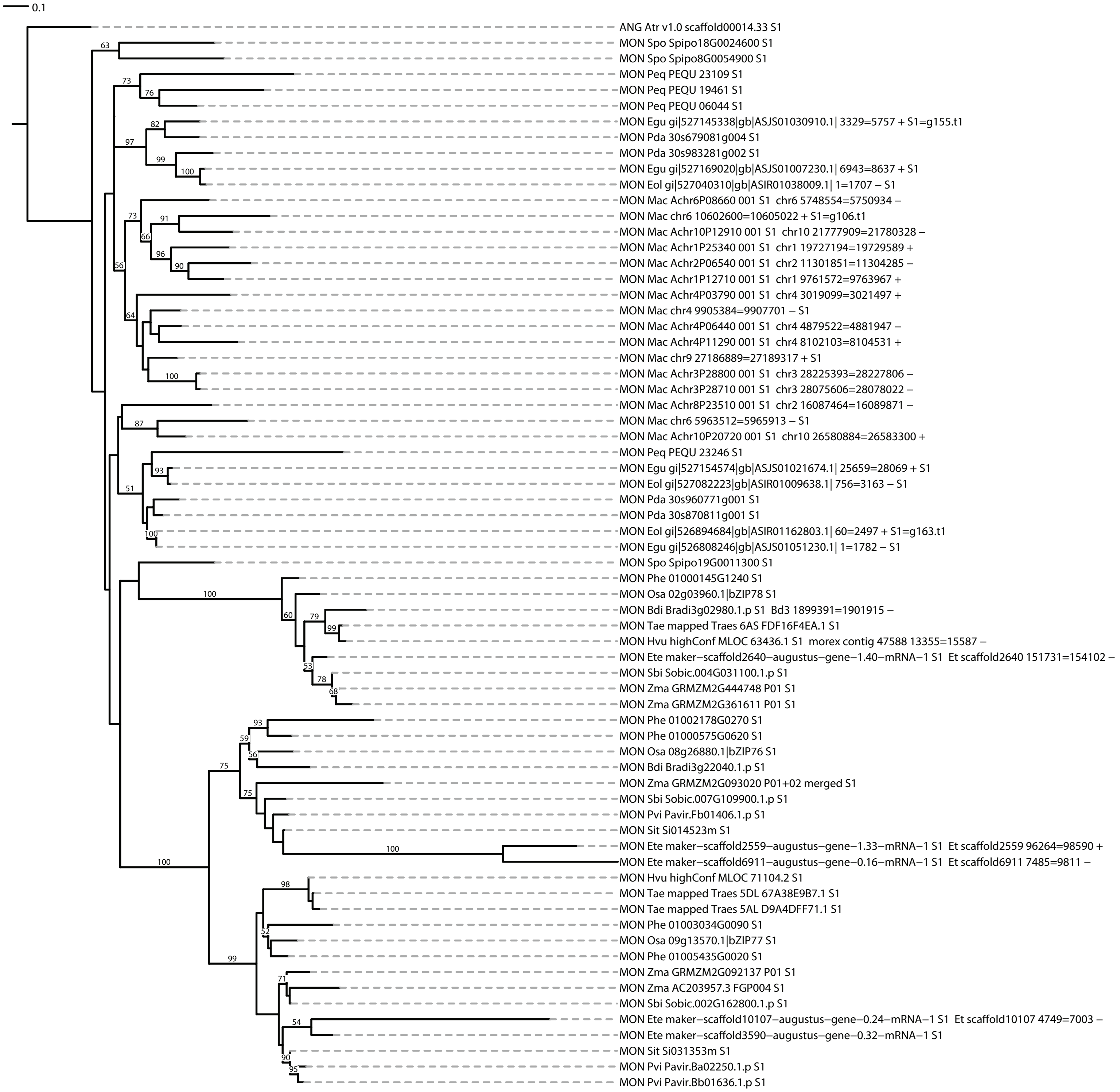

"The phylogeny of C/S1 bZIP transcription factors reveals a shared algal ancestry and the pre-angiosperm translational regulation of S1 transcripts". Peviani A., Lastdrager J., Hanson J., Snel B.

Figure S3: Phylogenetic trees of proto-C/S, cII, C, and S bZIPs from green plants.

Tree 1. proto-C/S, cII, C, and S bZIPs (annotated sequences only).

Tree 2. proto-C/S, cII, C, and S bZIPs (annotated+predicted sequences).

Tree 3. C bZIPs (annotated sequences only).

Tree 4. C bZIPs (annotated+predicted sequences).

Tree 5. S bZIPs (annotated sequences only).

Tree 6. S bZIPs (annotated+predicted sequences).

Tree 1. proto-C/S, cII, C, and S bZIPs (annotated only)

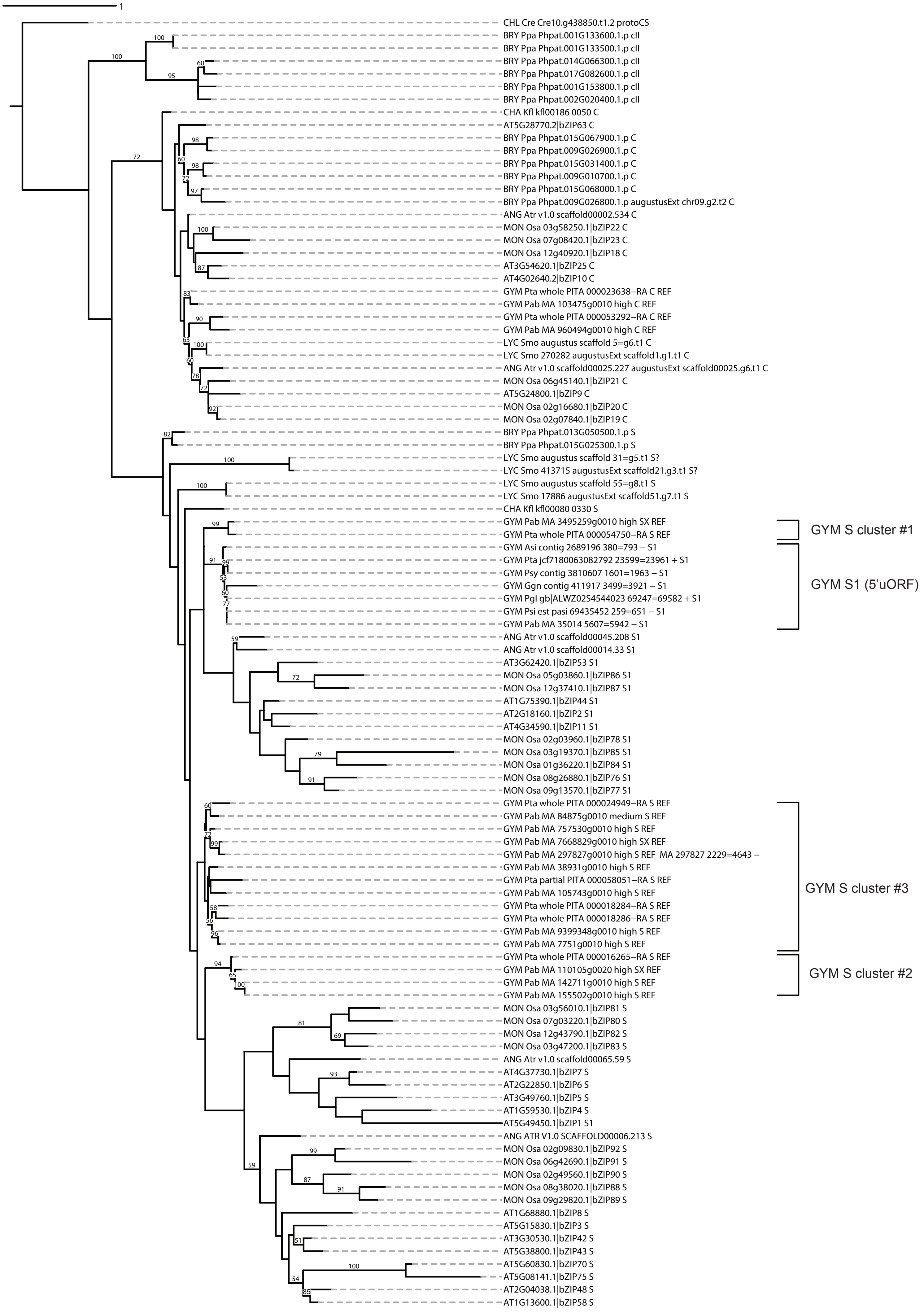

Tree 2. proto-C/S, cII, C, and S bZIPs (annotated + predicted)

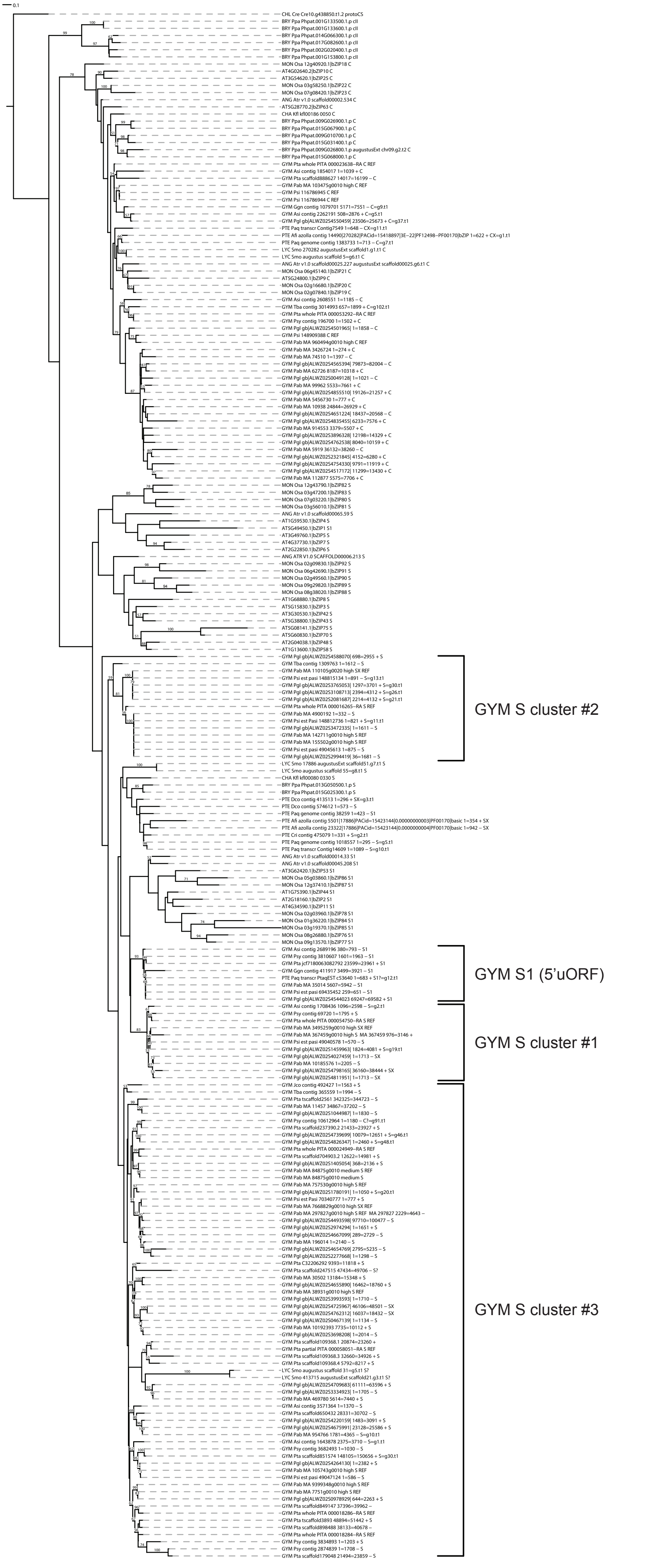

Tree 3. C bZIPs (annotated only)

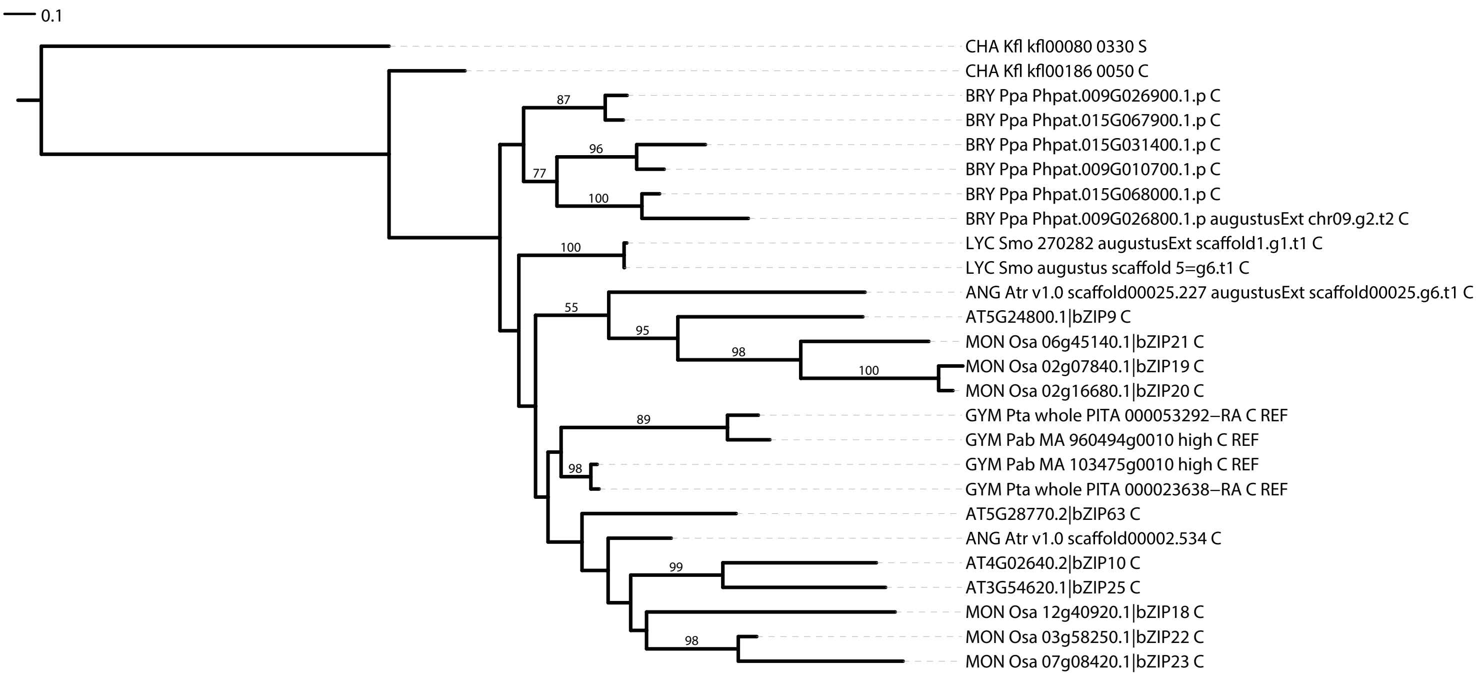

Tree 4. C bZIPs (annotated + predicted)

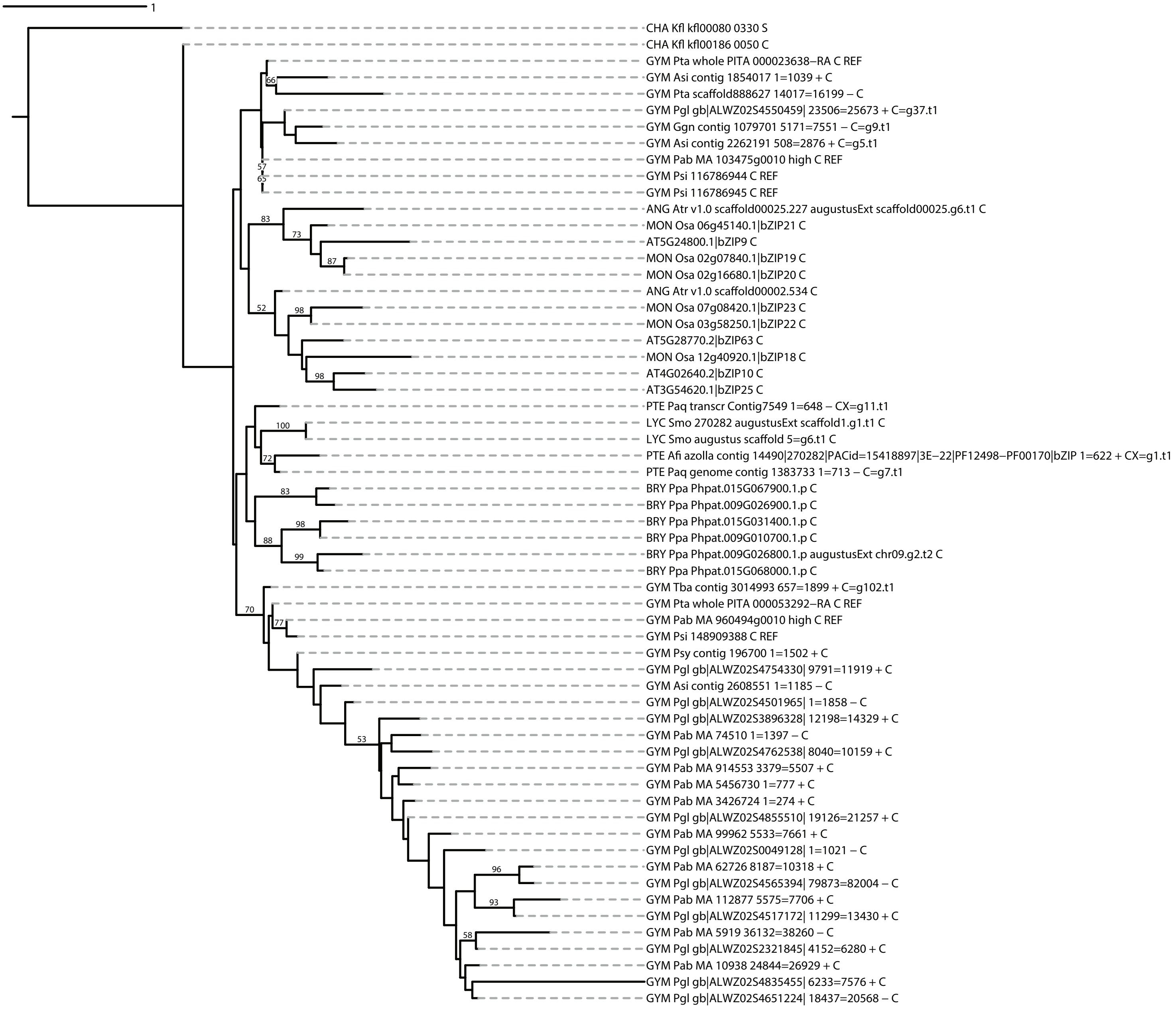

Tree 5. S bZIPs (annotated only)

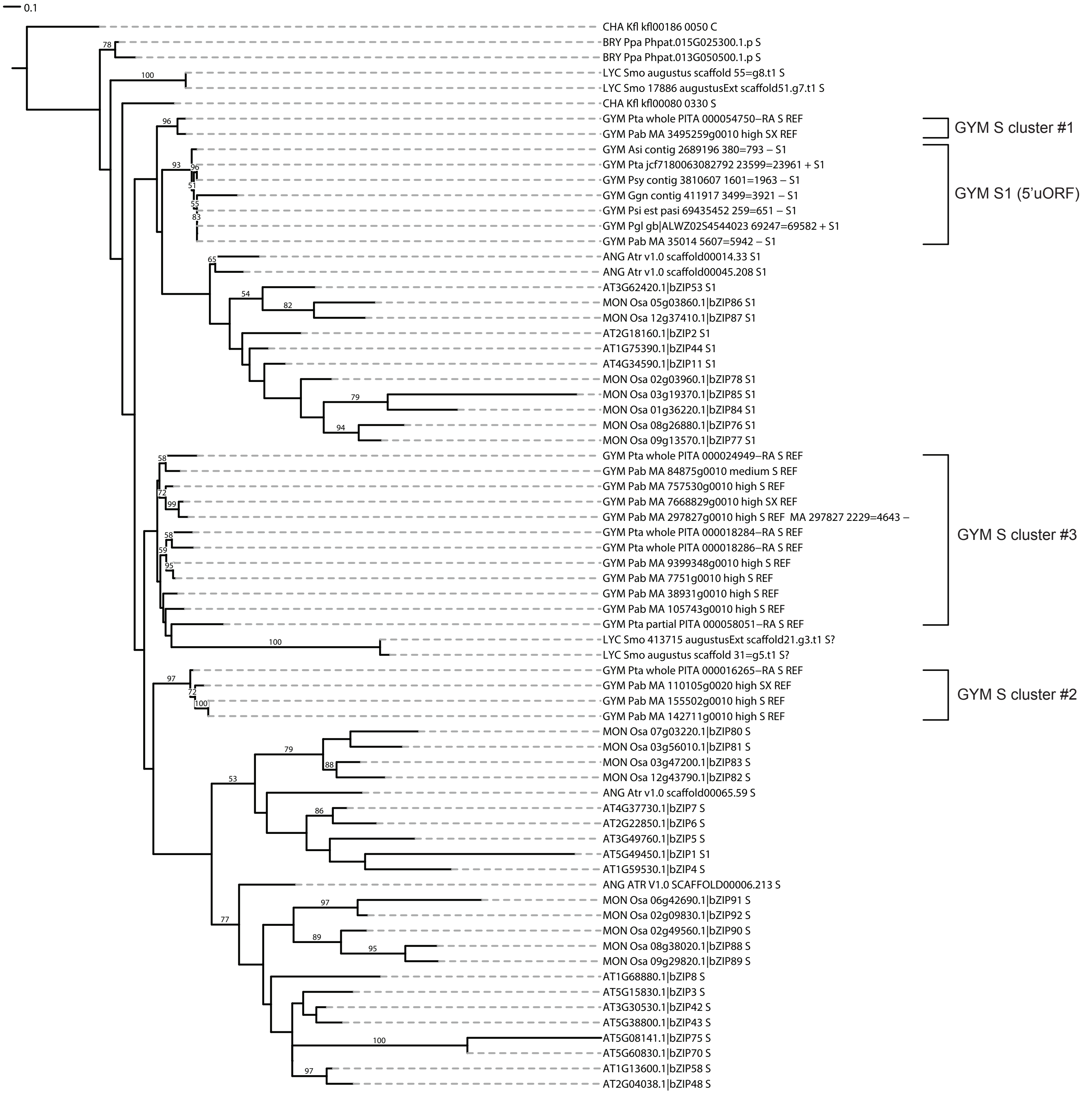

Tree 6. S bZIPs (annotated + predicted)

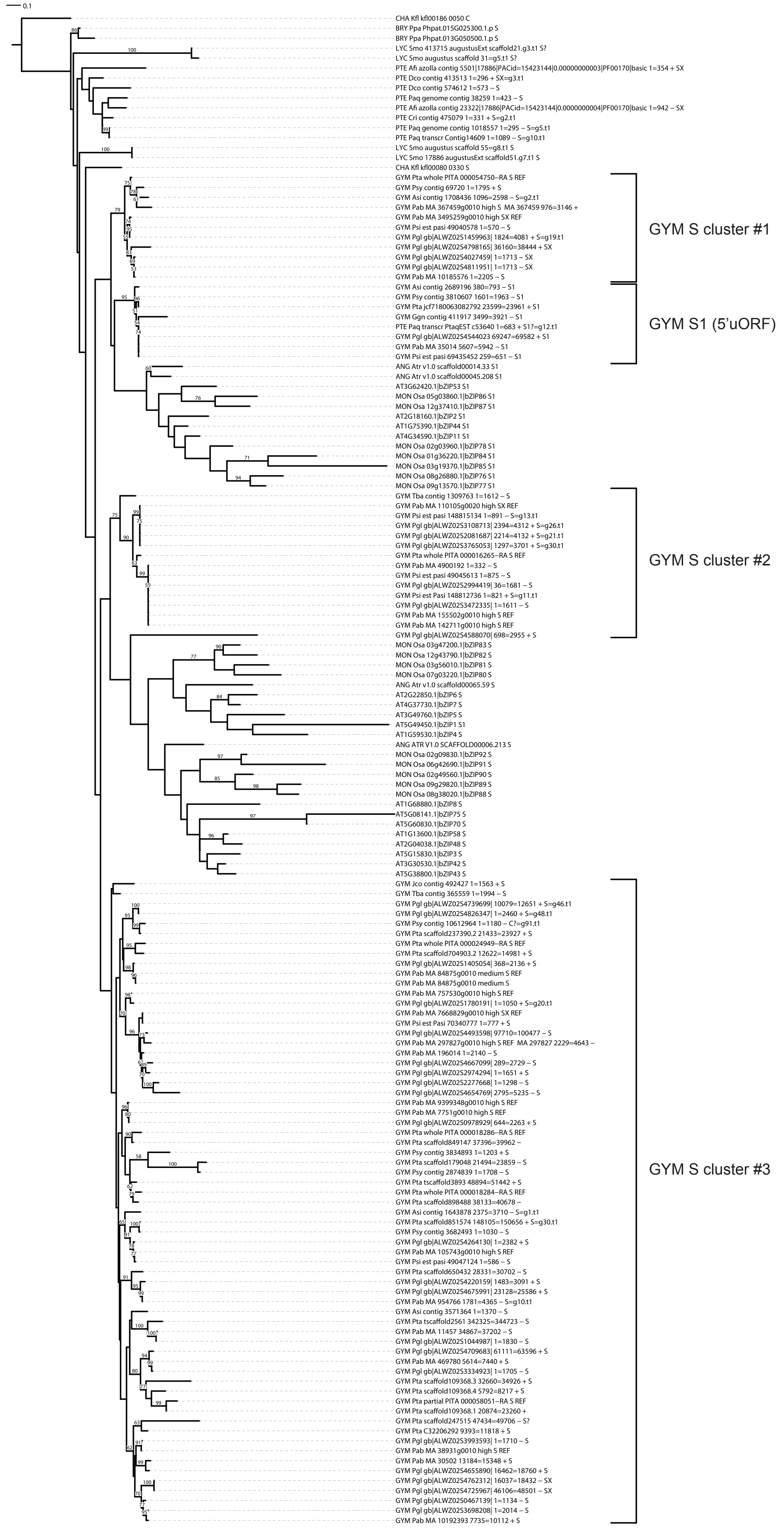

Supplement: Supplementary Information [file srep30444-s1.pdf]
